# Supplementary material for: Synthesis, Antimicrobial, and Antibiofilm Activities of Some Novel 7-Methoxyquinoline Derivatives Bearing Sulfonamide Moiety against Urinary Tract Infection-Causing Pathogenic Microbes
Source: Int J Mol Sci. 2023 May 18;24(10):8933. doi: 10.3390/ijms24108933 (PMC10219129; doi:10.3390/ijms24108933)
Supplement: Supplementary file 1 [file ijms-24-08933-s001.zip › ijms-2360752-supplementary.pdf]

Supplementary Data

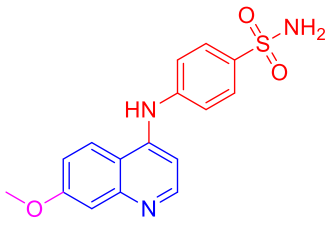

Compound 2

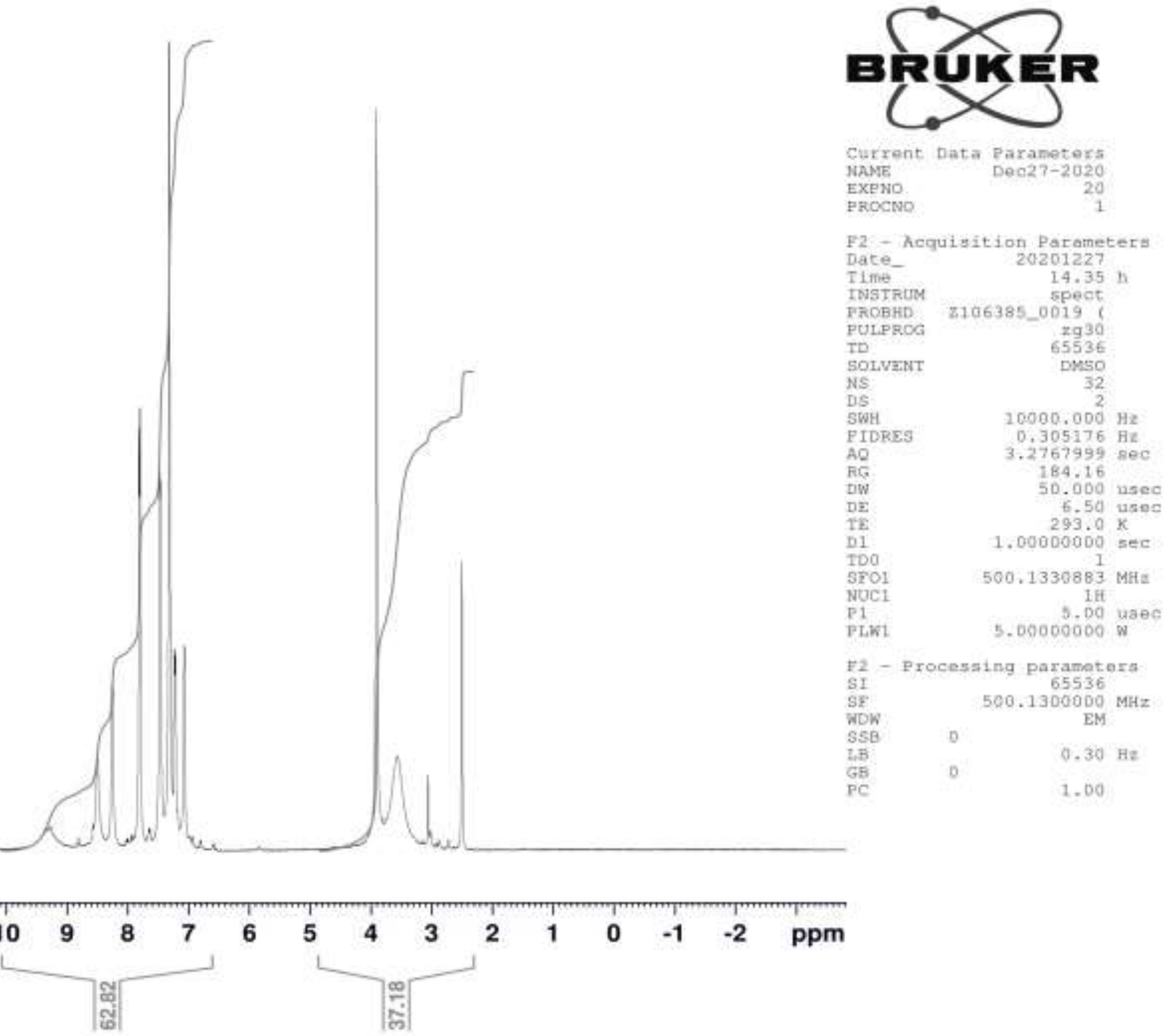

1- C13

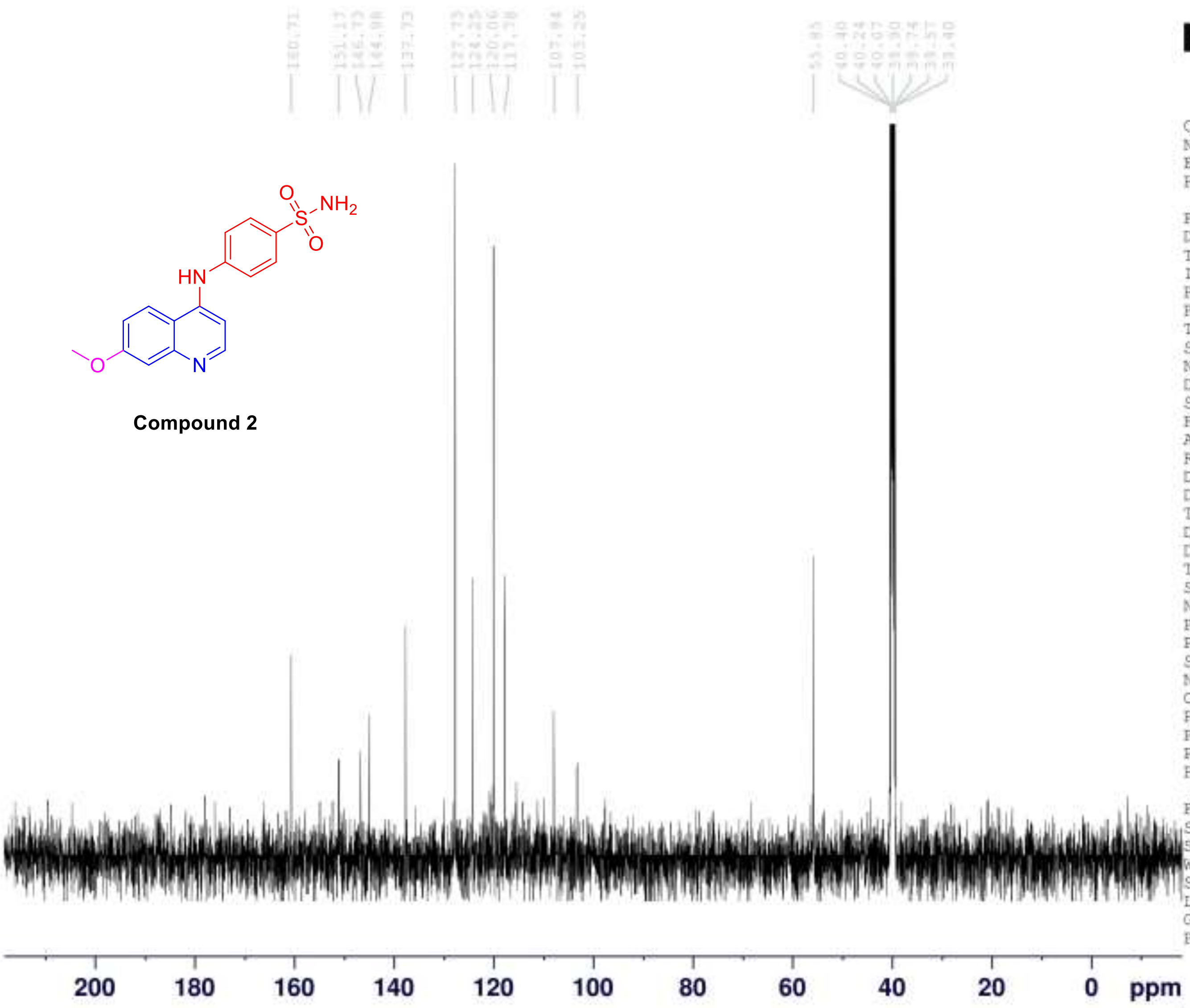


```
Current Data Parameters
NAME              Dec27-2020
EXPNO                     21
PROCNO                     1

F2 - Acquisition Parameters
Date_               20201227
Time                 15.56 h
INSTRUM                spect
PROBHD      Z106385_0019 (
PULPROG              zgpg30
TD                    65536
SOLVENT                DMSO
NS                     1500
DS                        4
SWH              29761.904 Hz
FIDRES            0.908261 Hz
AQ               1.1010048 sec
RG                  184.16
DW                  16.800 usec
DE                    6.50 usec
TE                   293.0 K
D1              2.00000000 sec
D11             0.03000000 sec
TD0                       1
SFO1           125.7703643 MHz
NUC1                    13C
P1                    10.00 usec
PLW1           27.00000000 W
SFO2           500.1320005 MHz
NUC2                     1H
CPDPRG[2            waltz16
PCPD2                80.00 usec
PLW2            5.00000000 W
PLW12           0.01953100 W
PLW13           0.00982410 W

F2 - Processing parameters
SI                    32768
SF             125.7577885 MHz
WDW                      EM
SSB           0
LB                     1.00 Hz
GB            0
PC                     1.40
```

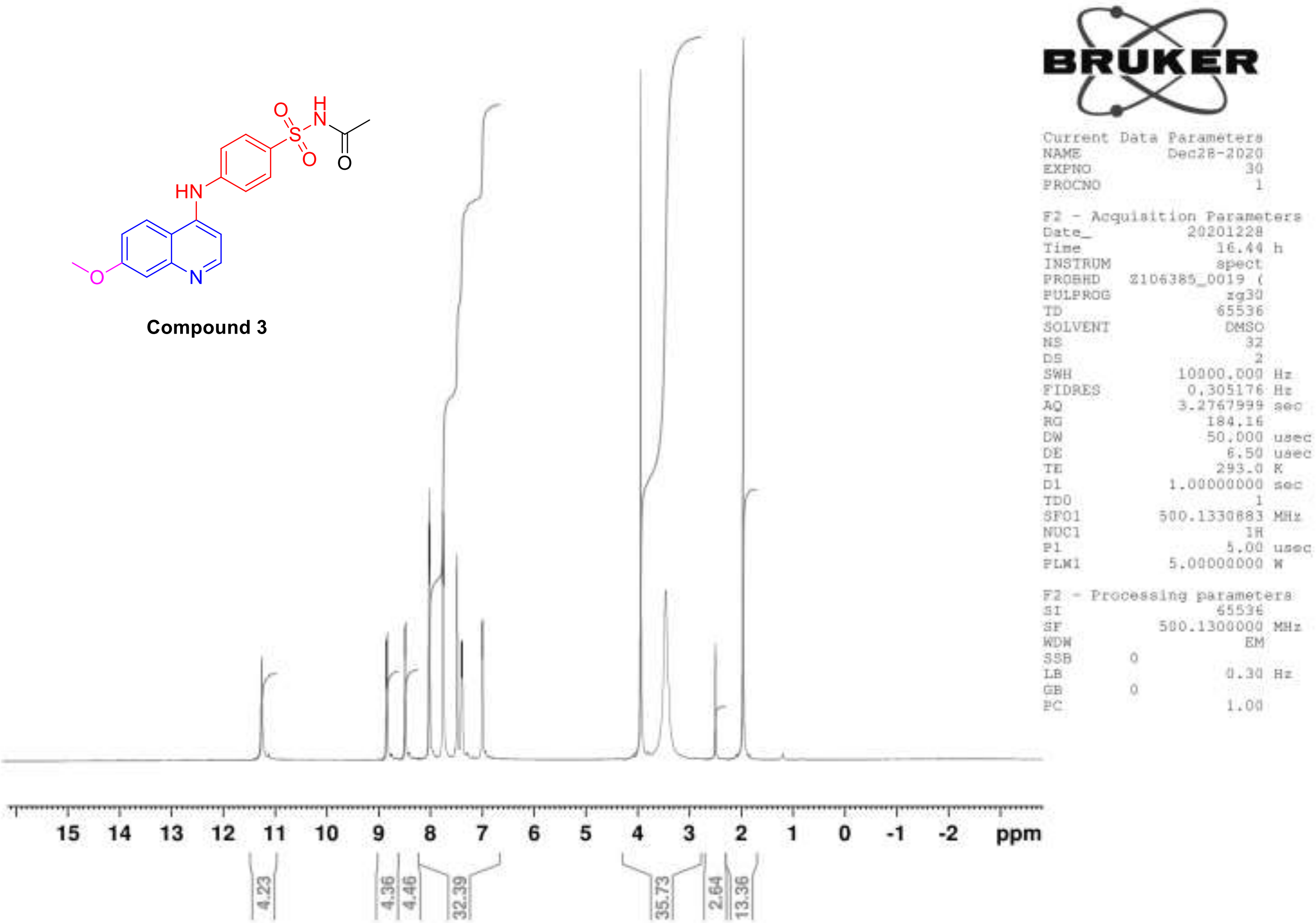
O
S
O
H
N
O
HN
O
N
Compound 3
BRUKER
Current Data Parameters
NAME Dec28-2020
EXPNO 30
PROCNO 1
F2 - Acquisition Parameters
Date_ 20201228
Time 16.44 h
INSTRUM spect
PROBHD Z106385_0019 (
PULPROG zg30
TD 65536
SOLVENT DMSO
NS 32
DS 2
SWH 10000.000 Hz
FIDRES 0.305176 Hz
AQ 3.2767999 sec
RG 184.16
DW 50.000 usec
DE 6.50 usec
TE 293.0 K
D1 1.00000000 sec
TD0 1
SFO1 500.1330883 MHz
NUC1 1H
P1 5.00 usec
PLW1 5.00000000 W
F2 - Processing parameters
SI 65536
SF 500.1300000 MHz
WDW EM
SSB 0
LB 0.30 Hz
GB 0
PC 1.00
15 14 13 12 11 10 9 8 7 6 5 4 3 2 1 0 -1 -2 ppm
4.23
4.36
4.46
32.39
35.73
2.64
13.36

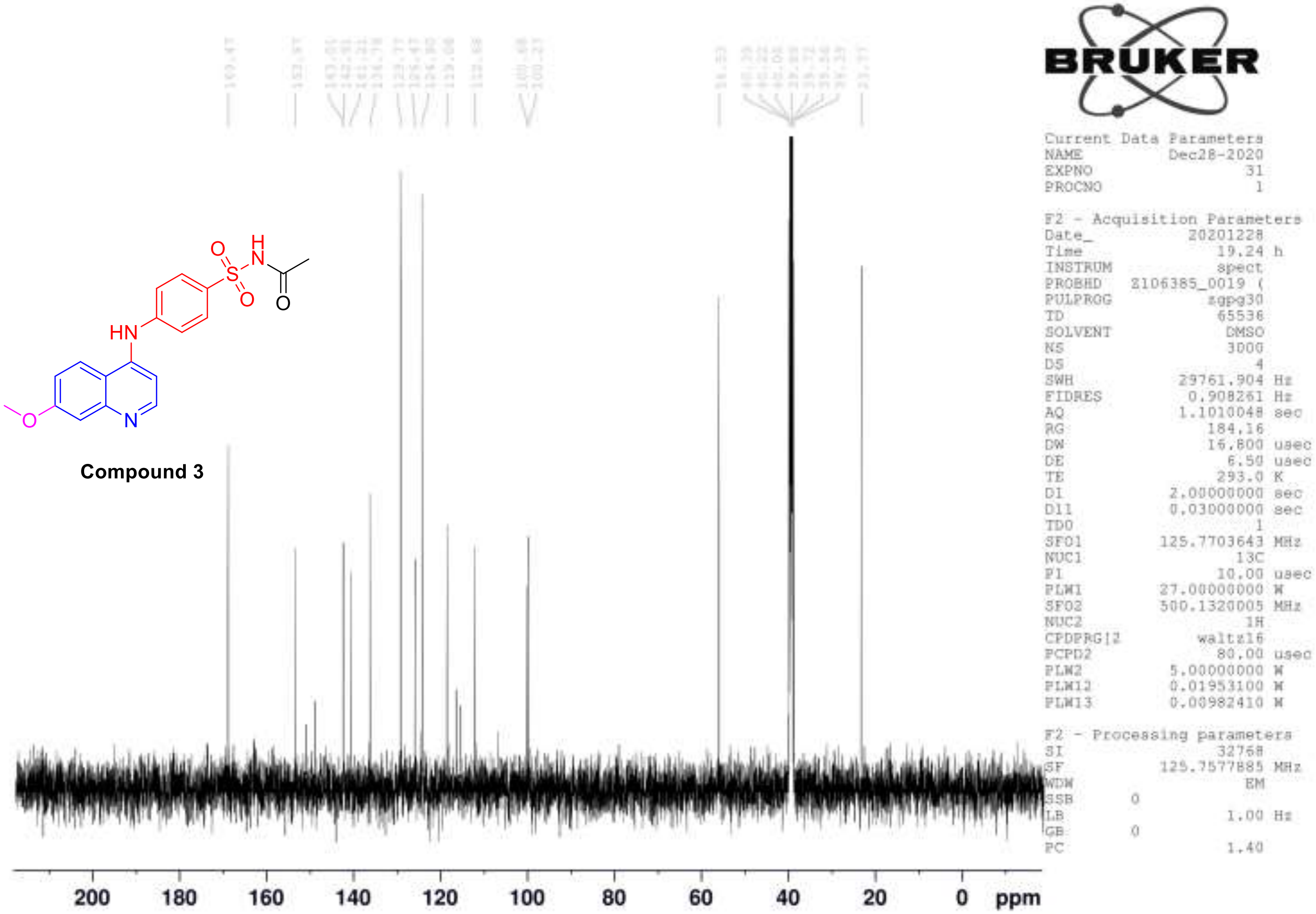

HN
N
O
S
O
H
N
O
O
Compound 3
BRUKER
Current Data Parameters
NAME Dec28-2020
EXPNO 31
PROCNO 1
F2 - Acquisition Parameters
Date_ 20201228
Time 19.24 h
INSTRUM spect
PROBHD Z106385_0019 (
PULPROG zgpg30
TD 65536
SOLVENT DMSO
NS 3000
DS 4
SWH 29761.904 Hz
FIDRES 0.908261 Hz
AQ 1.1010048 sec
RG 184.16
DW 16.800 usec
DE 6.50 usec
TE 293.0 K
D1 2.00000000 sec
D11 0.03000000 sec
TD0 1
SFO1 125.7703643 MHz
NUC1 13C
P1 10.00 usec
PLW1 27.00000000 W
SFO2 500.1320005 MHz
NUC2 1H
CPDPRG[2 waltz16
PCPD2 80.00 usec
PLW2 5.00000000 W
PLW12 0.01953100 W
PLW13 0.00982410 W
F2 - Processing parameters
SI 32768
SF 125.7577885 MHz
WDW EM
SSB 0
LB 1.00 Hz
GB 0
PC 1.40
200 180 160 140 120 100 80 60 40 20 0 ppm

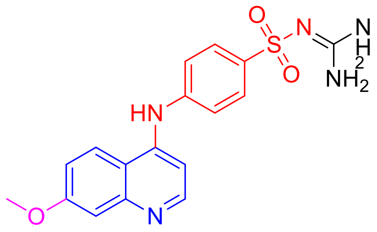
O
S
O
N
N
H
NH2
HN
N
O

Compound 4

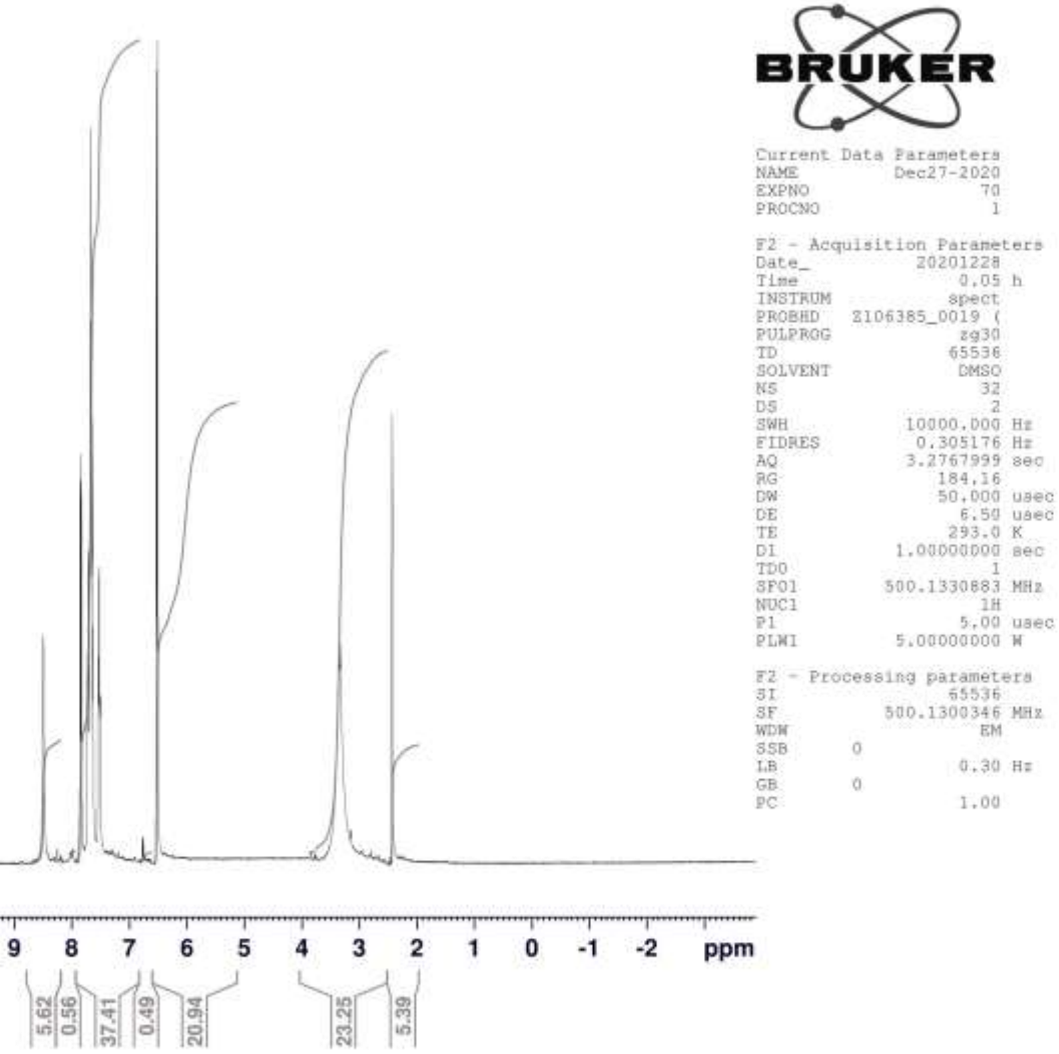
Current Data Parameters
NAME Dec27-2020
EXPNO 70
PROCNO 1
F2 - Acquisition Parameters
Date_ 20201228
Time 0.05 h
INSTRUM spect
PROBHD Z106385_0019 (
PULPROG zg30
TD 65536
SOLVENT DMSO
NS 32
DS 2
SWH 10000.000 Hz
FIDRES 0.305176 Hz
AQ 3.2767999 sec
RG 184.16
DW 50.000 usec
DE 6.50 usec
TE 293.0 K
D1 1.00000000 sec
TD0 1
SFO1 500.1330883 MHz
NUC1 1H
P1 5.00 usec
PLW1 5.00000000 W
F2 - Processing parameters
SI 65536
SF 500.1300346 MHz
WDW EM
SSB 0
LB 0.30 Hz
GB 0
PC 1.00
15 14 13 12 11 10 9 8 7 6 5 4 3 2 1 0 -1 -2 ppm
5.11
5.62
0.56
37.41
0.49
20.94
23.25
5.39

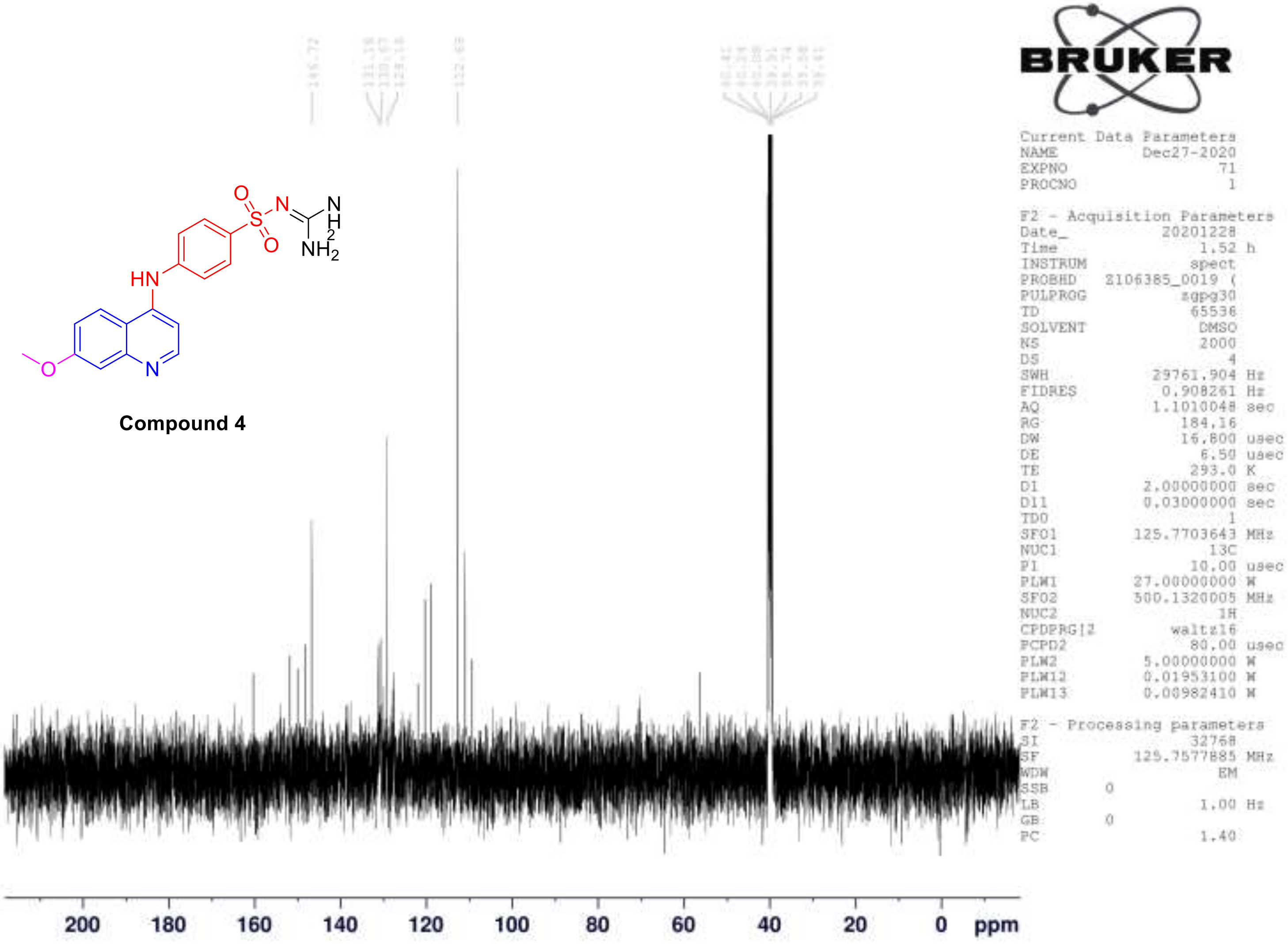
HN
N
O
S
O
N
NH2
NH2
Compound 4
BRUKER
Current Data Parameters
NAME Dec27-2020
EXPNO 71
PROCNO 1
F2 - Acquisition Parameters
Date_ 20201228
Time 1.52 h
INSTRUM spect
PROBHD Z106385_0019 (
PULPROG zgpg30
TD 65536
SOLVENT DMSO
NS 2000
DS 4
SWH 29761.904 Hz
FIDRES 0.908261 Hz
AQ 1.1010048 sec
RG 184.16
DW 16.800 usec
DE 6.50 usec
TE 293.0 K
D1 2.00000000 sec
D11 0.03000000 sec
TD0 1
SFO1 125.7703643 MHz
NUC1 13C
P1 10.00 usec
PLW1 27.00000000 W
SFO2 500.1320005 MHz
NUC2 1H
CPDPRG[2 waltz16
PCPD2 80.00 usec
PLW2 5.00000000 W
PLW12 0.01953100 W
PLW13 0.00982410 W
F2 - Processing parameters
SI 32768
SF 125.7577885 MHz
WDW EM
SSB 0
LB 1.00 Hz
GB 0
PC 1.40
200 180 160 140 120 100 80 60 40 20 0 ppm

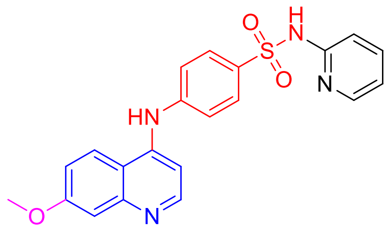
O
H
N
S
O
N
HN
O
N

Compound 5

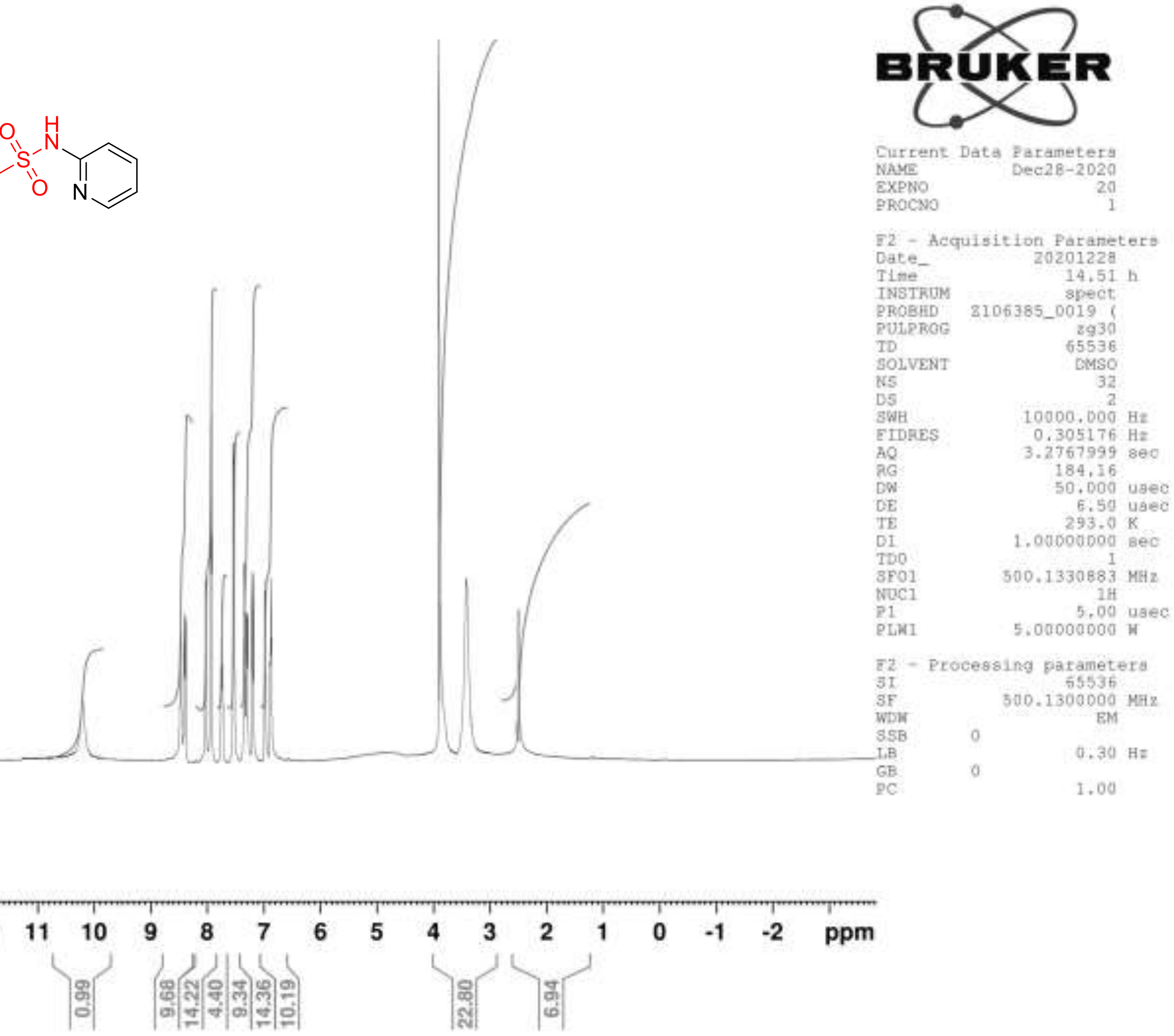
BRUKER
Current Data Parameters
NAME Dec28-2020
EXPNO 20
PROCNO 1
F2 - Acquisition Parameters
Date_ 20201228
Time 14.51 h
INSTRUM spect
PROBHD Z106385_0019 (
PULPROG zg30
TD 65536
SOLVENT DMSO
NS 32
DS 2
SWH 10000.000 Hz
FIDRES 0.305176 Hz
AQ 3.2767999 sec
RG 184.16
DW 50.000 usec
DE 6.50 usec
TE 293.0 K
D1 1.00000000 sec
TD0 1
SFO1 500.1330883 MHz
NUC1 1H
P1 5.00 usec
PLW1 5.00000000 W
F2 - Processing parameters
SI 65536
SF 500.1300000 MHz
WDW EM
SSB 0
LB 0.30 Hz
GB 0
PC 1.00
11 10 9 8 7 6 5 4 3 2 1 0 -1 -2 ppm
0.99
9.68
14.22
4.40
9.34
14.36
10.19
22.80
6.94

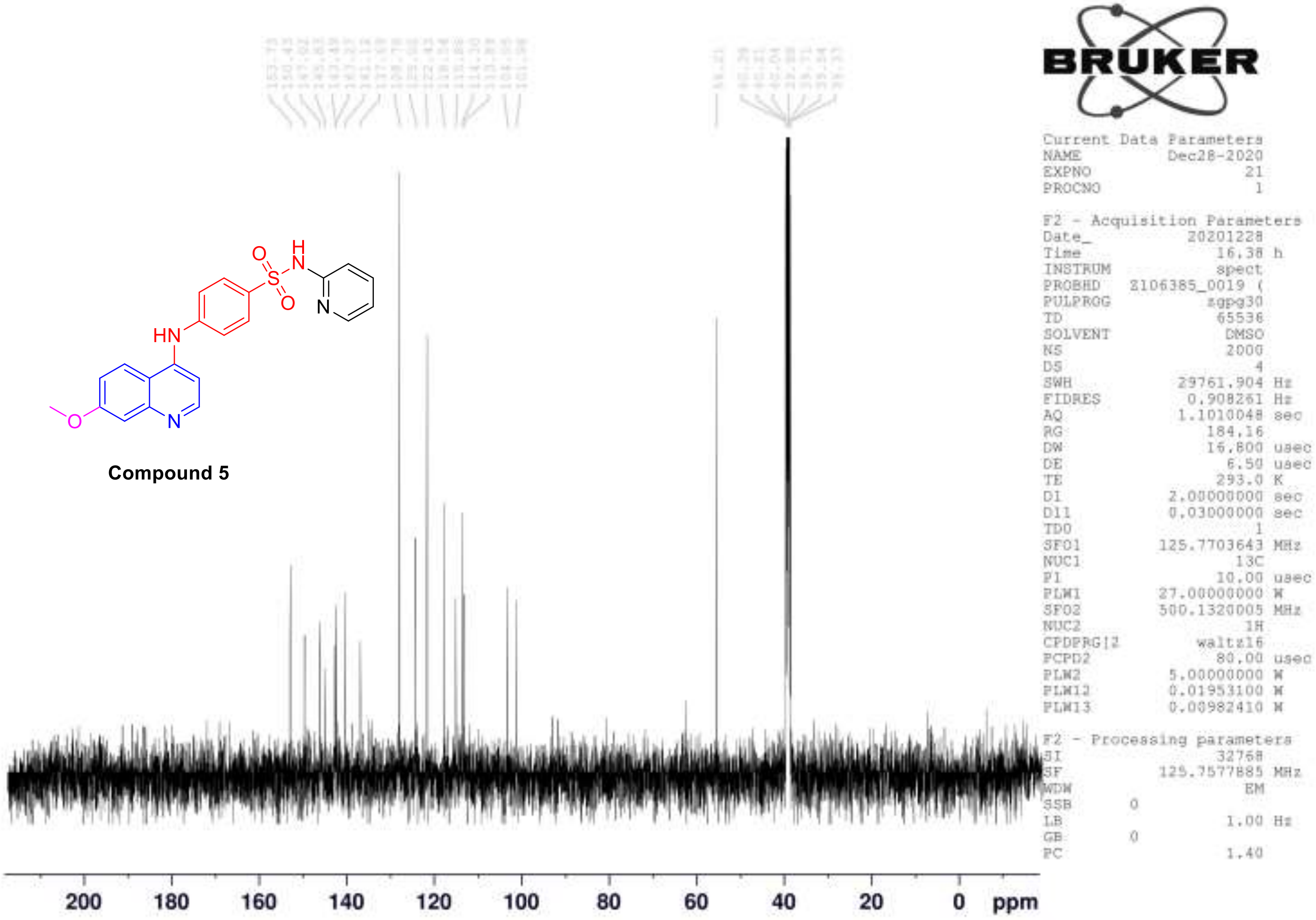

HN
H
N
O
S
O
N
O
N
BRUKER
Current Data Parameters
NAME Dec28-2020
EXPNO 21
PROCNO 1
F2 - Acquisition Parameters
Date_ 20201228
Time 16.38 h
INSTRUM spect
PROBHD Z106385_0019 (
PULPROG zgpg30
TD 65536
SOLVENT DMSO
NS 2000
DS 4
SWH 29761.904 Hz
FIDRES 0.908261 Hz
AQ 1.1010048 sec
RG 184.16
DW 16.800 usec
DE 6.50 usec
TE 293.0 K
D1 2.00000000 sec
D11 0.03000000 sec
TD0 1
SFO1 125.7703643 MHz
NUC1 13C
P1 10.00 usec
PLW1 27.00000000 W
SFO2 500.1320005 MHz
NUC2 1H
CPDPRG[2 waltz16
PCPD2 80.00 usec
PLW2 5.00000000 W
PLW12 0.01953100 W
PLW13 0.00982410 W
F2 - Processing parameters
SI 32768
SF 125.7577885 MHz
WDW EM
SSB 0
LB 1.00 Hz
GB 0
PC 1.40
200 180 160 140 120 100 80 60 40 20 0 ppm

Compound 5

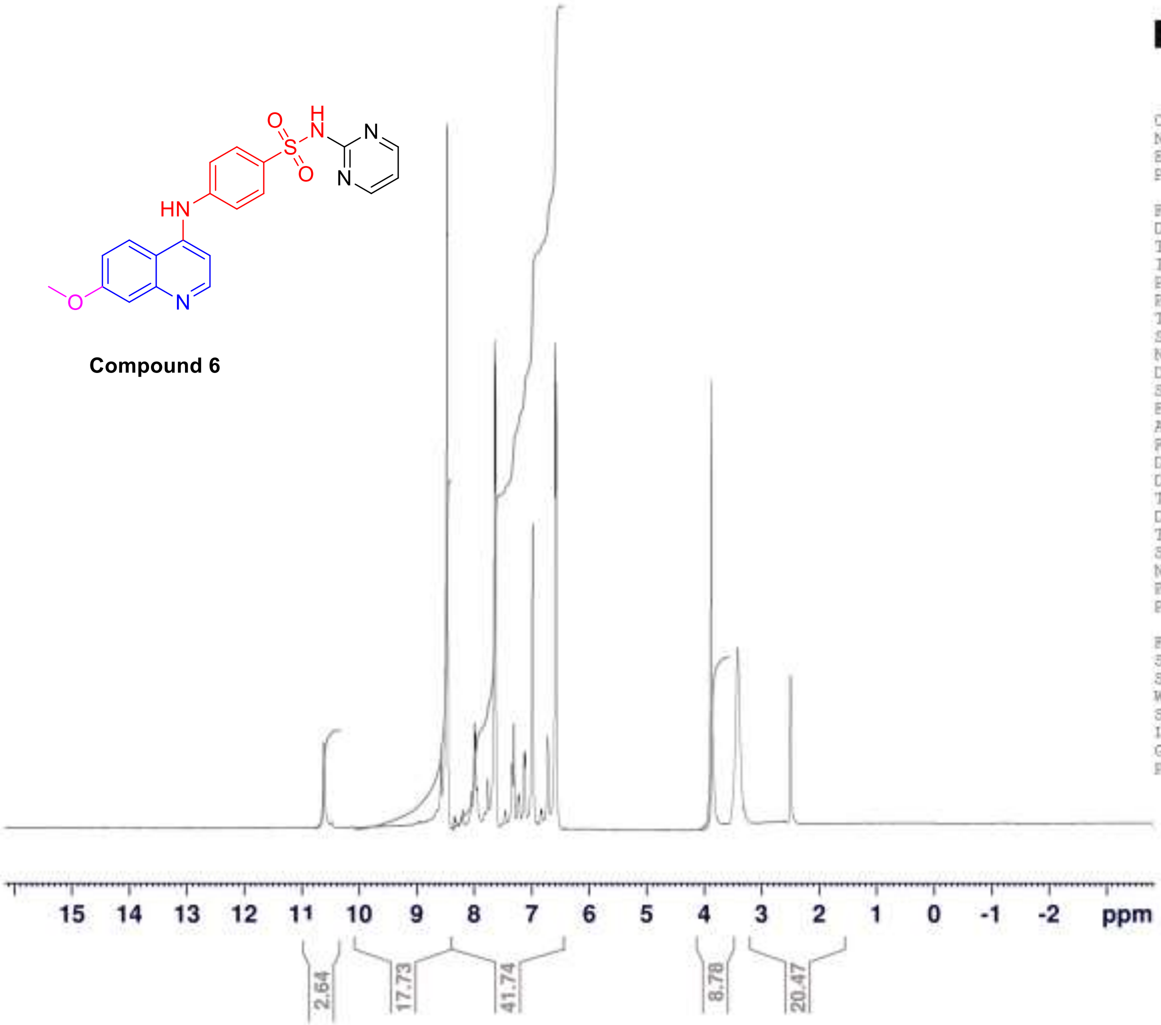

Compound 6

BRUKER

Current Data Parameters
NAME Dec27-2020
EXPNO 60
PROCNO 1

F2 - Acquisition Parameters
Date_ 20201227
Time 22.12 h
INSTRUM spect
PROBHD Z106385_0019 (
PULPROG zg30
TD 65536
SOLVENT DMSO
NS 32
DS 2
SWH 10000.000 Hz
FIDRES 0.305176 Hz
AQ 3.2767999 sec
RG 131.78
DW 50.000 usec
DE 6.50 usec
TE 293.0 K
D1 1.00000000 sec
TD0 1
SFO1 500.1330883 MHz
NUC1 1H
P1 5.00 usec
PLW1 5.00000000 W

F2 - Processing parameters
SI 65536
SF 500.1300000 MHz
WDW EM
SSB 0
LB 0.30 Hz
GB 0
PC 1.00

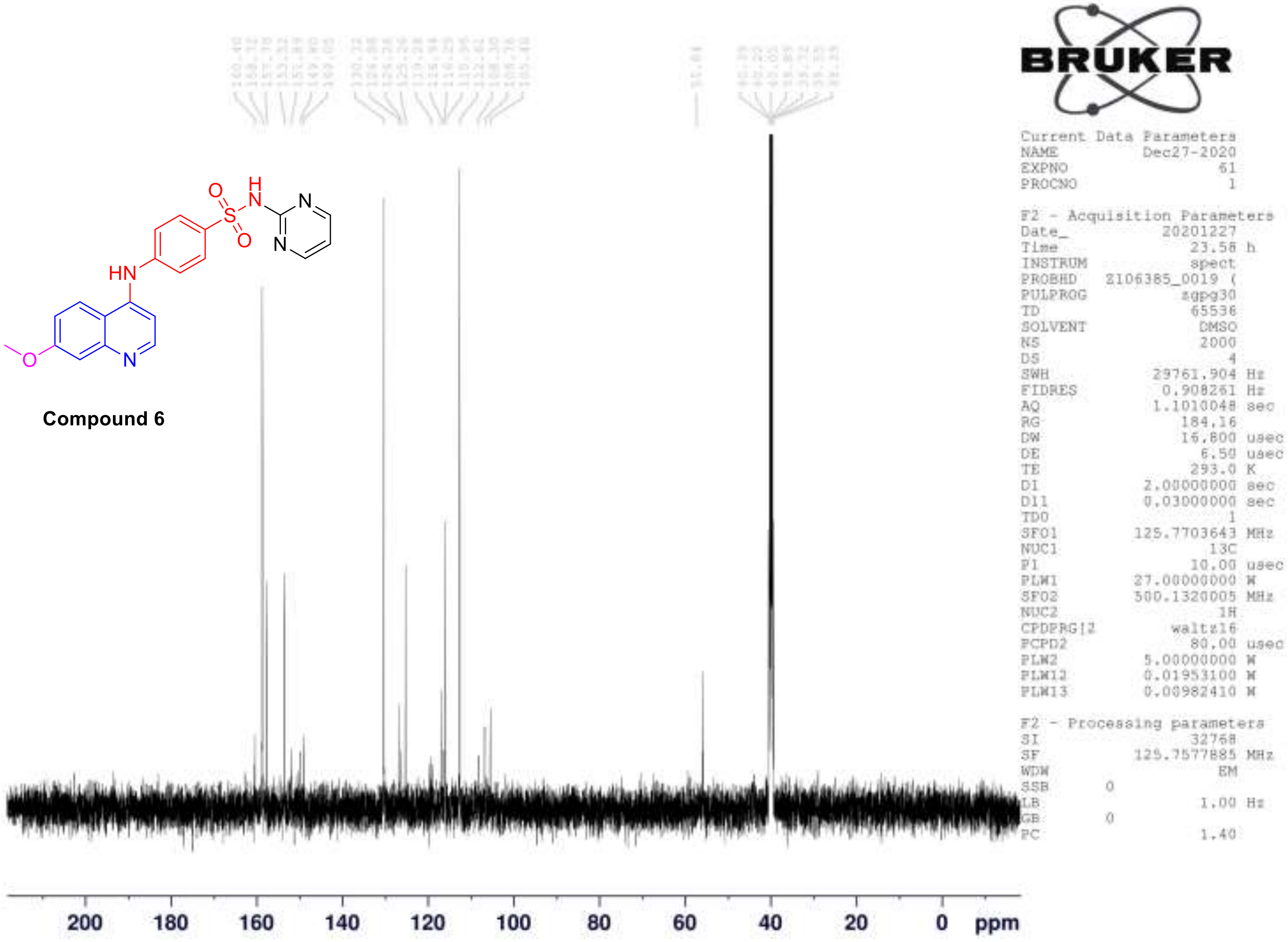

Compound 6
HN
N
O
S
O
H
N
N
N
BRUKER
Current Data Parameters
NAME Dec27-2020
EXPNO 61
PROCNO 1
F2 - Acquisition Parameters
Date_ 20201227
Time 23.58 h
INSTRUM spect
PROBHD Z106385_0019 (
PULPROG zgpg30
TD 65536
SOLVENT DMSO
NS 2000
DS 4
SWH 29761.904 Hz
FIDRES 0.908261 Hz
AQ 1.1010048 sec
RG 184.16
DW 16.800 usec
DE 6.50 usec
TE 293.0 K
D1 2.00000000 sec
D11 0.03000000 sec
TD0 1
SFO1 125.7703643 MHz
NUC1 13C
P1 10.00 usec
PLW1 27.00000000 W
SFO2 500.1320005 MHz
NUC2 1H
CPDPRG[2 waltz16
PCPD2 80.00 usec
PLW2 5.00000000 W
PLW12 0.01953100 W
PLW13 0.00982410 W
F2 - Processing parameters
SI 32768
SF 125.7577885 MHz
WDW EM
SSB 0
LB 1.00 Hz
GB 0
PC 1.40
200 180 160 140 120 100 80 60 40 20 0 ppm

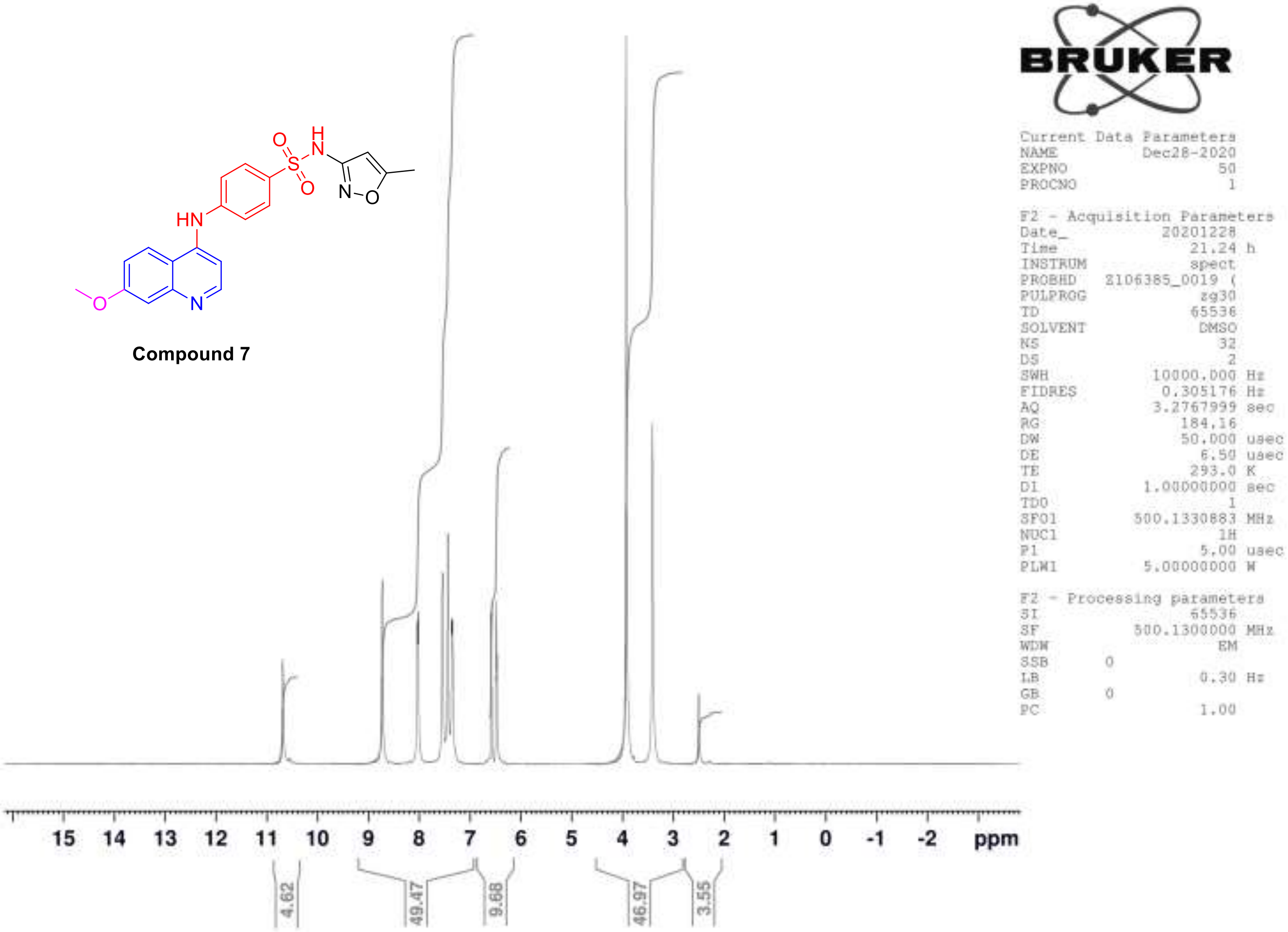
O
S
O
H
N
N
O
HN
O
N
Compound 7
BRUKER
Current Data Parameters
NAME Dec28-2020
EXPNO 50
PROCNO 1
F2 - Acquisition Parameters
Date_ 20201228
Time 21.24 h
INSTRUM spect
PROBHD Z106385_0019 (
PULPROG zg30
TD 65536
SOLVENT DMSO
NS 32
DS 2
SWH 10000.000 Hz
FIDRES 0.305176 Hz
AQ 3.2767999 sec
RG 184.16
DW 50.000 usec
DE 6.50 usec
TE 293.0 K
D1 1.00000000 sec
TD0 1
SFO1 500.1330883 MHz
NUC1 1H
P1 5.00 usec
PLW1 5.00000000 W
F2 - Processing parameters
SI 65536
SF 500.1300000 MHz
WDW EM
SSB 0
LB 0.30 Hz
GB 0
PC 1.00
15 14 13 12 11 10 9 8 7 6 5 4 3 2 1 0 -1 -2 ppm
4.62
49.47
9.68
46.97
3.55

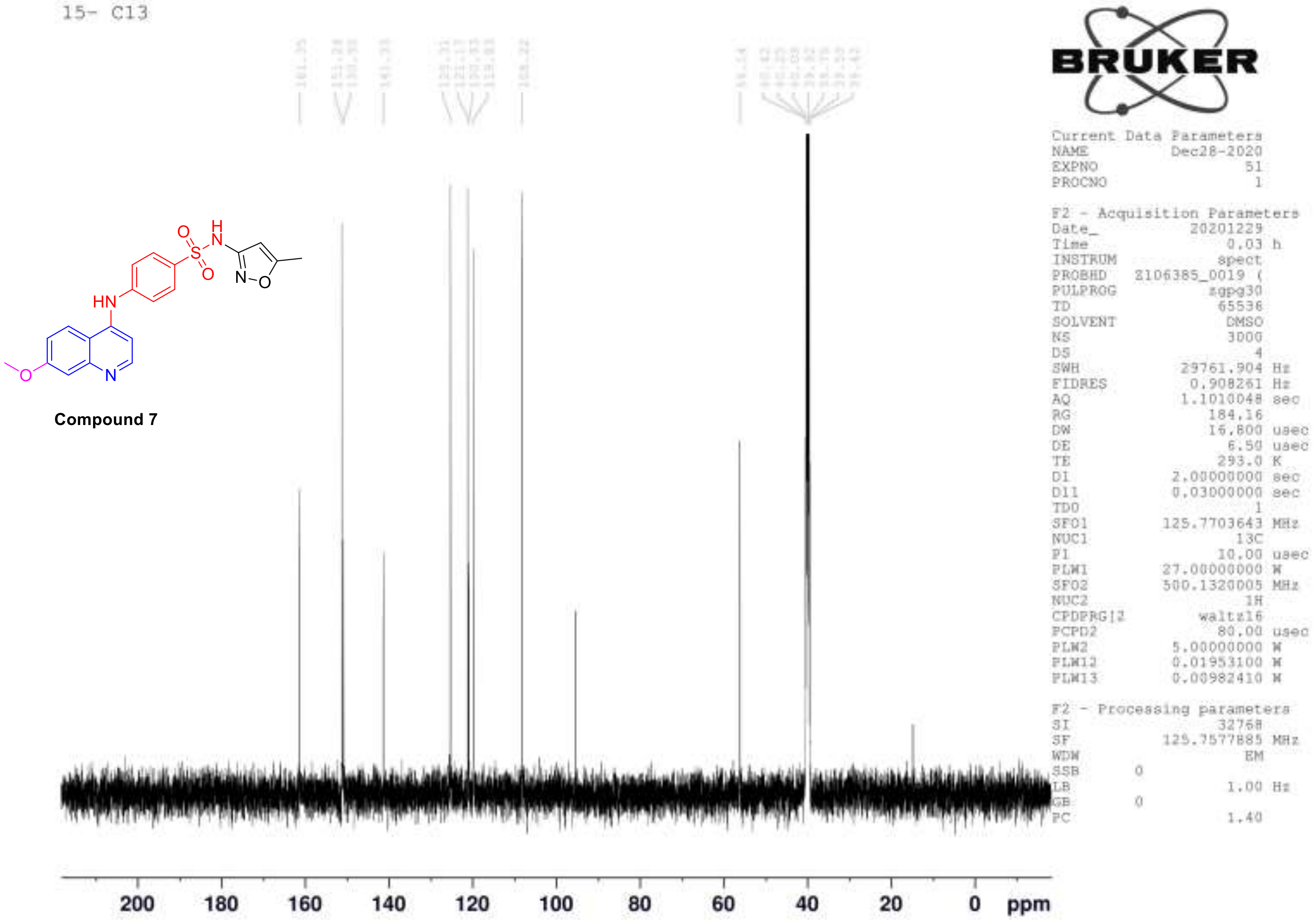

15- C13
HN
N
O
S
O
O
H
N
N-O
BRUKER
Current Data Parameters
NAME Dec28-2020
EXPNO 51
PROCNO 1
F2 - Acquisition Parameters
Date_ 20201229
Time 0.03 h
INSTRUM spect
PROBHD Z106385_0019 (
PULPROG zgpg30
TD 65536
SOLVENT DMSO
NS 3000
DS 4
SWH 29761.904 Hz
FIDRES 0.908261 Hz
AQ 1.1010048 sec
RG 184.16
DW 16.800 usec
DE 6.50 usec
TE 293.0 K
D1 2.00000000 sec
D11 0.03000000 sec
TD0 1
SFO1 125.7703643 MHz
NUC1 13C
P1 10.00 usec
PLW1 27.00000000 W
SFO2 500.1320005 MHz
NUC2 1H
CPDPRG[2 waltz16
PCPD2 80.00 usec
PLW2 5.00000000 W
PLW12 0.01953100 W
PLW13 0.00982410 W
F2 - Processing parameters
SI 32768
SF 125.7577885 MHz
WDW EM
SSB 0
LB 1.00 Hz
GB 0
PC 1.40
200
180
160
140
120
100
80
60
40
20
0
ppm

Compound 7

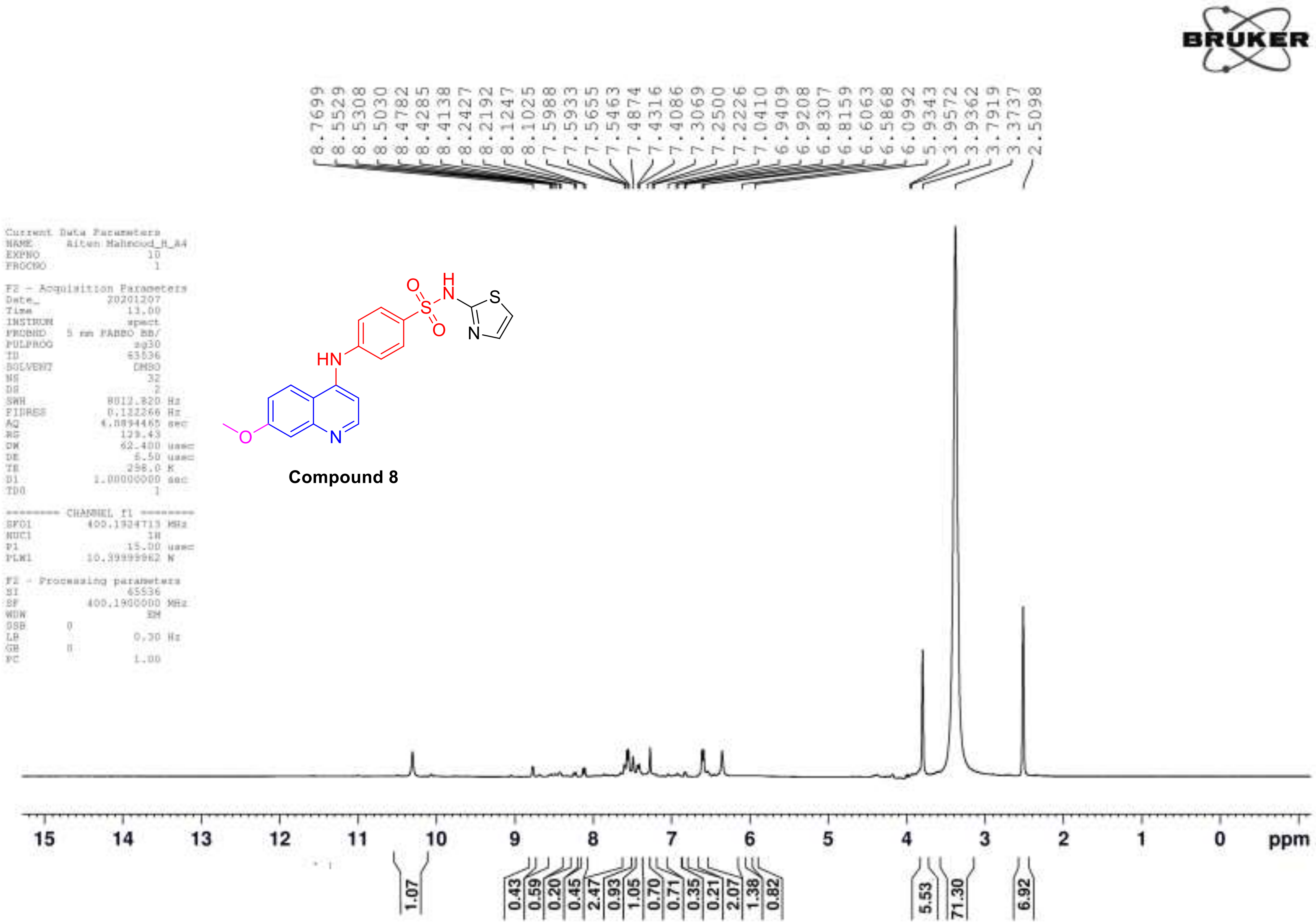
BRUKER
8.7699
8.5529
8.5308
8.5030
8.4782
8.4285
8.4138
8.2427
8.2192
8.1247
8.1025
7.5988
7.5933
7.5655
7.5463
7.4874
7.4316
7.4086
7.3069
7.2500
7.2226
7.0410
6.9409
6.9208
6.8307
6.8159
6.6063
6.5868
6.0992
5.9343
3.9572
3.9362
3.7919
3.3737
2.5098
Current Data Parameters
NAME Aiten Mahmood_H_A4
EXPNO 10
PROCNO 1
F2 - Acquisition Parameters
Date_ 20201207
Time 13.00
INSTRUM spect
PROBHD 5 mm PABBO BB/
PULPROG zg30
TD 65536
SOLVENT DMSO
NS 32
DS 2
SWH 8012.820 Hz
FIDRES 0.122266 Hz
AQ 4.0894465 sec
RG 129.43
DW 62.400 usec
DE 6.50 usec
TE 298.0 K
D1 1.00000000 sec
TD0 1
======== CHANNEL f1 ========
SFO1 400.1924713 MHz
NUC1 1H
P1 15.00 usec
PLW1 10.39999962 W
F2 - Processing parameters
SI 65536
SF 400.1900000 MHz
WDW EM
SSB 0
LB 0.30 Hz
GB 0
PC 1.00
O
H
N
S
O
N
HN
O
N
Compound 8
15
14
13
12
11
10
9
8
7
6
5
4
3
2
1
0
ppm
1.07
0.43
0.59
0.20
0.45
2.47
0.93
1.05
0.70
0.71
0.35
0.21
2.07
1.38
0.82
5.53
71.30
6.92

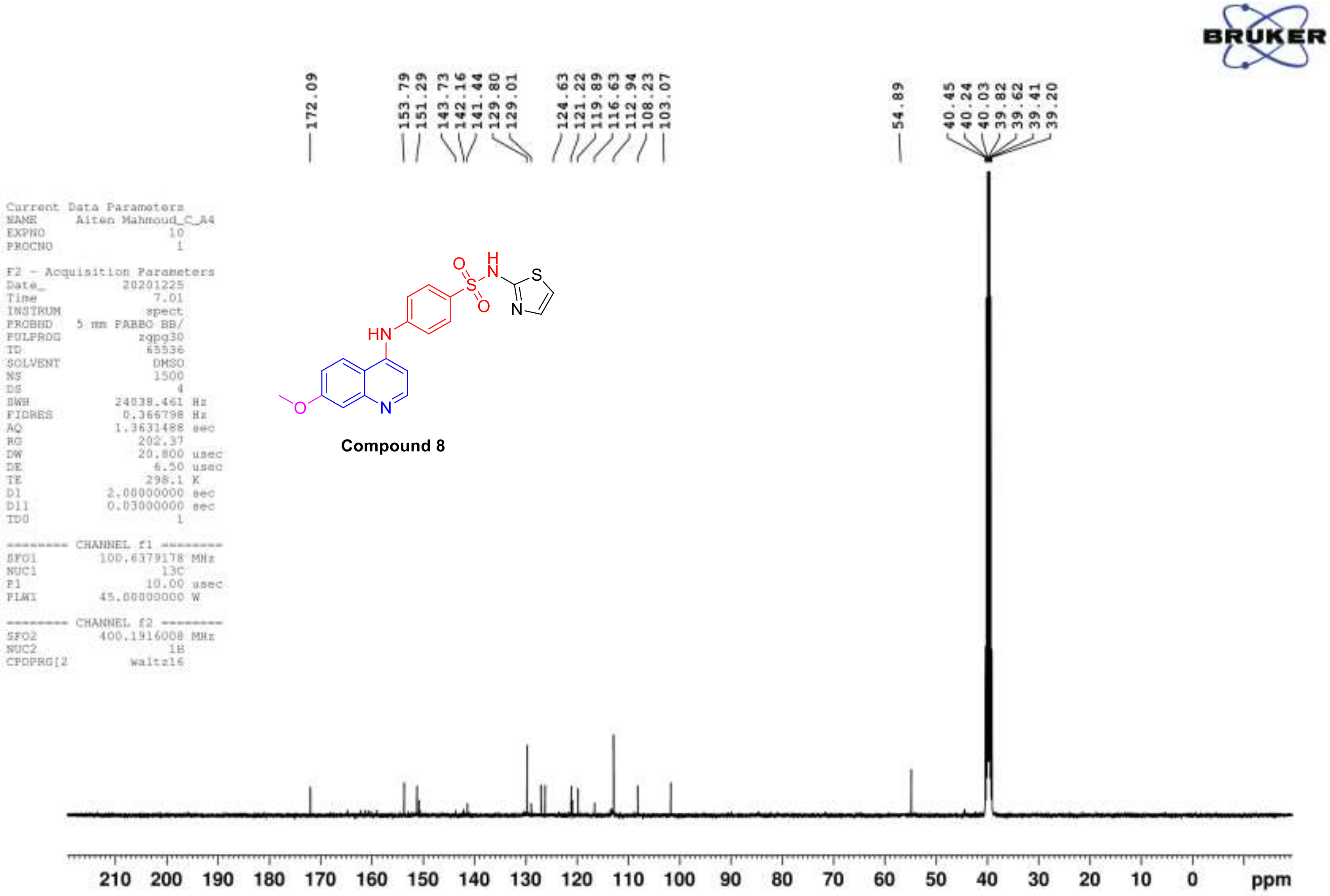
172.09
153.79
151.29
143.73
142.16
141.44
129.80
129.01
124.63
121.22
119.89
116.63
112.94
108.23
103.07
54.89
40.45
40.24
40.03
39.82
39.62
39.41
39.20
BRUKER
Current Data Parameters
NAME Aiten Mahmoud_C_A4
EXPNO 10
PROCNO 1
F2 - Acquisition Parameters
Date_ 20201225
Time 7.01
INSTRUM spect
PROBHD 5 mm PABBO BB/
PULPROG zgpg30
TD 65536
SOLVENT DMSO
NS 1500
DS 4
SWH 24038.461 Hz
FIDRES 0.366798 Hz
AQ 1.3631488 sec
RG 202.37
DW 20.800 usec
DE 6.50 usec
TE 298.1 K
D1 2.00000000 sec
D11 0.03000000 sec
TD0 1
======== CHANNEL f1 ========
SFO1 100.6379178 MHz
NUC1 13C
P1 10.00 usec
PLW1 45.00000000 W
======== CHANNEL f2 ========
SFO2 400.1916008 MHz
NUC2 1H
CPDPRG[2 waltz16
Compound 8
210 200 190 180 170 160 150 140 130 120 110 100 90 80 70 60 50 40 30 20 10 0 ppm

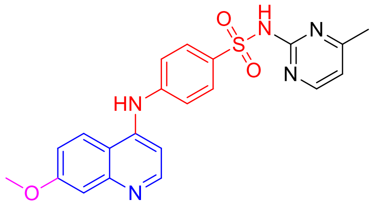
O
S
O
H
N
N
N
HN
N
O

Compound 9

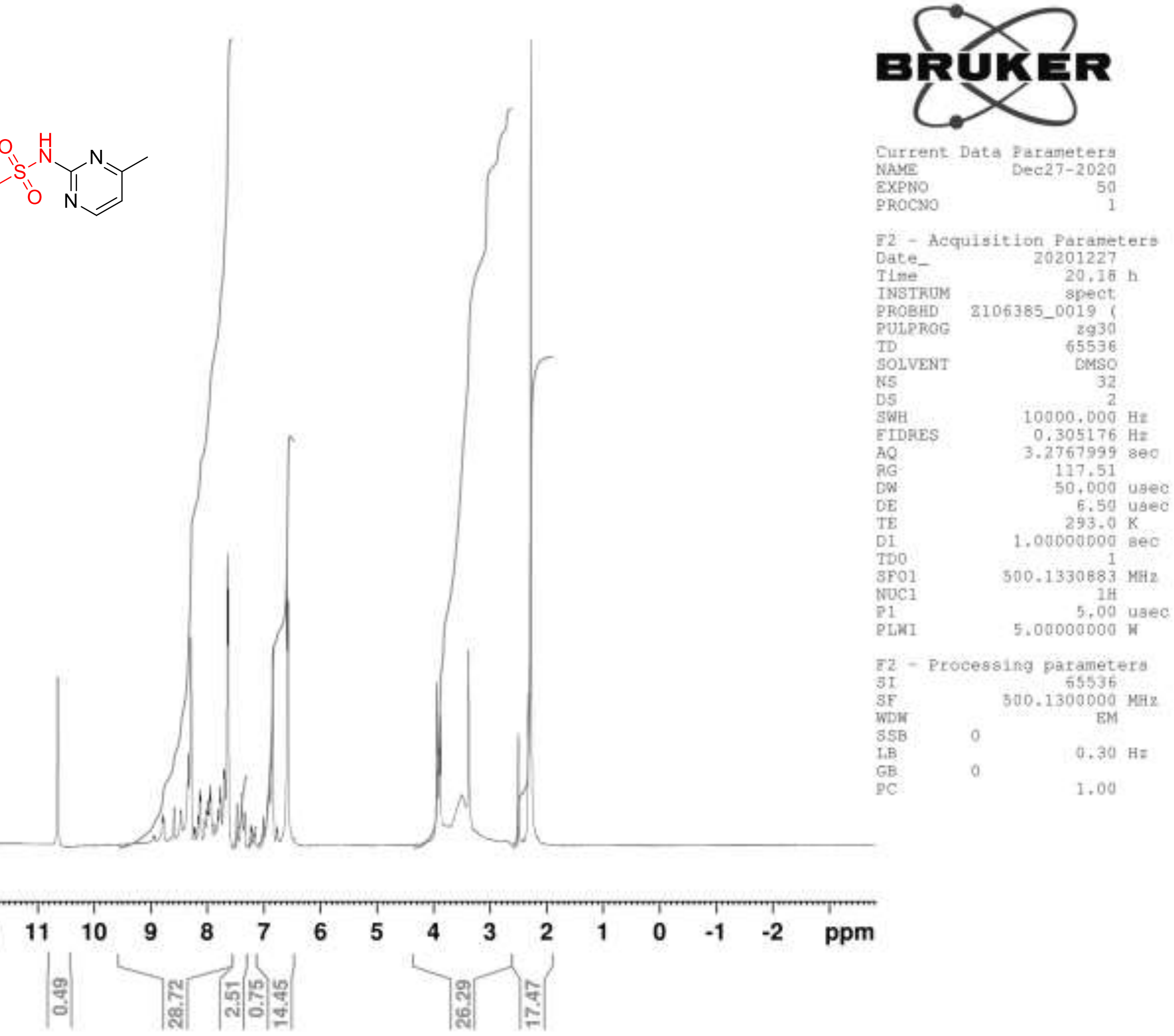
BRUKER
Current Data Parameters
NAME Dec27-2020
EXPNO 50
PROCNO 1
F2 - Acquisition Parameters
Date_ 20201227
Time 20.18 h
INSTRUM spect
PROBHD Z106385_0019 (
PULPROG zg30
TD 65536
SOLVENT DMSO
NS 32
DS 2
SWH 10000.000 Hz
FIDRES 0.305176 Hz
AQ 3.2767999 sec
RG 117.51
DW 50.000 usec
DE 6.50 usec
TE 293.0 K
D1 1.00000000 sec
TD0 1
SFO1 500.1330883 MHz
NUC1 1H
P1 5.00 usec
PLW1 5.00000000 W
F2 - Processing parameters
SI 65536
SF 500.1300000 MHz
WDW EM
SSB 0
LB 0.30 Hz
GB 0
PC 1.00
15 14 13 12 11 10 9 8 7 6 5 4 3 2 1 0 -1 -2 ppm
0.49
28.72
2.51
0.75
14.45
26.29
17.47

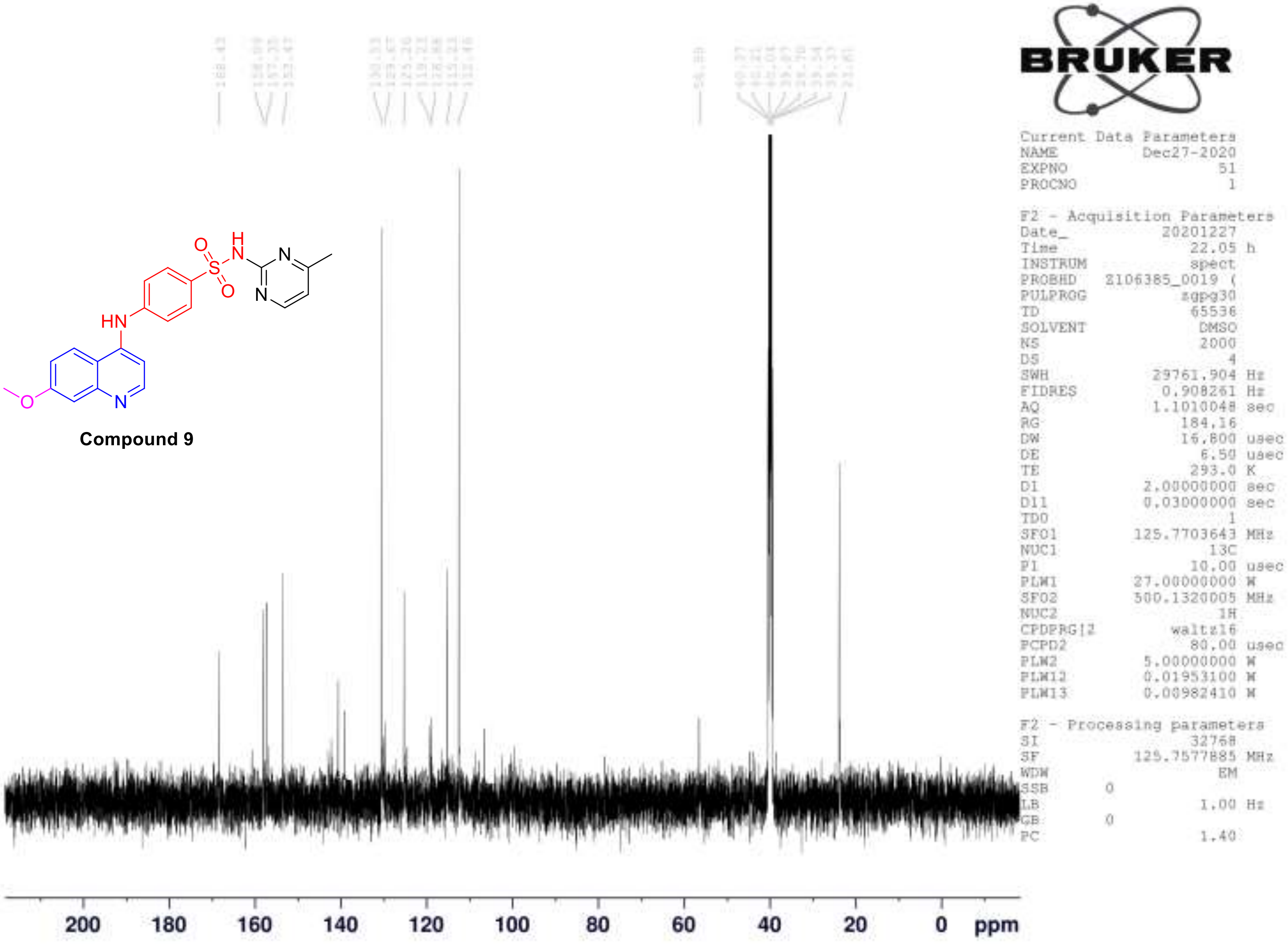

HN
O
S
O
H
N
N
N
N
O
Compound 9
BRUKER
Current Data Parameters
NAME Dec27-2020
EXPNO 51
PROCNO 1
F2 - Acquisition Parameters
Date_ 20201227
Time 22.05 h
INSTRUM spect
PROBHD Z106385_0019 (
PULPROG zgpg30
TD 65536
SOLVENT DMSO
NS 2000
DS 4
SWH 29761.904 Hz
FIDRES 0.908261 Hz
AQ 1.1010048 sec
RG 184.16
DW 16.800 usec
DE 6.50 usec
TE 293.0 K
D1 2.00000000 sec
D11 0.03000000 sec
TD0 1
SFO1 125.7703643 MHz
NUC1 13C
P1 10.00 usec
PLW1 27.00000000 W
SFO2 500.1320005 MHz
NUC2 1H
CPDPRG[2 waltz16
PCPD2 80.00 usec
PLW2 5.00000000 W
PLW12 0.01953100 W
PLW13 0.00982410 W
F2 - Processing parameters
SI 32768
SF 125.7577885 MHz
WDW EM
SSB 0
LB 1.00 Hz
GB 0
PC 1.40
200
180
160
140
120
100
80
60
40
20
0
ppm

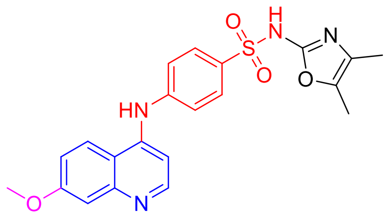
O
S
O
H
N
N
O
HN
O
N

Compound 10

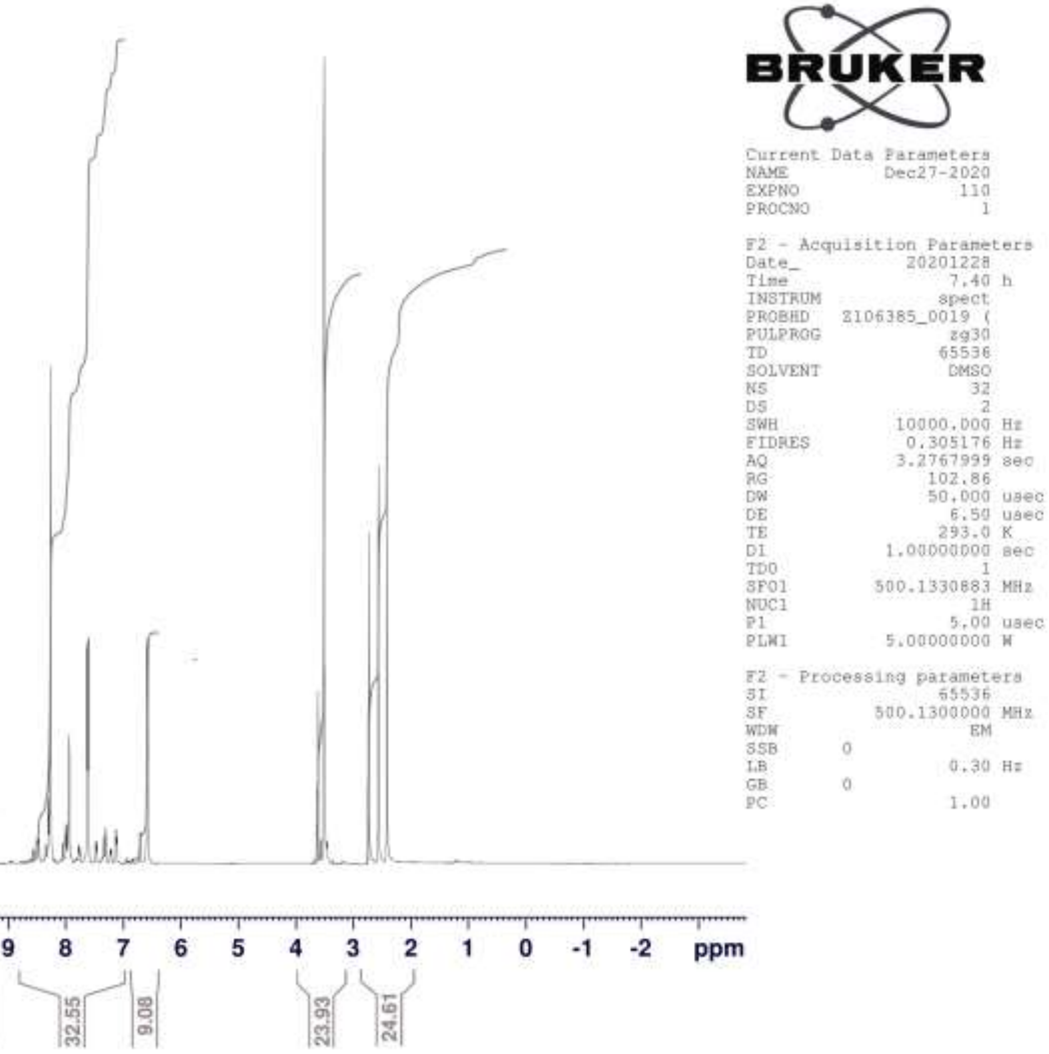
BRUKER
Current Data Parameters
NAME Dec27-2020
EXPNO 110
PROCNO 1
F2 - Acquisition Parameters
Date_ 20201228
Time 7.40 h
INSTRUM spect
PROBHD Z106385_0019 (
PULPROG zg30
TD 65536
SOLVENT DMSO
NS 32
DS 2
SWH 10000.000 Hz
FIDRES 0.305176 Hz
AQ 3.2767999 sec
RG 102.86
DW 50.000 usec
DE 6.50 usec
TE 293.0 K
D1 1.00000000 sec
TD0 1
SFO1 500.1330883 MHz
NUC1 1H
P1 5.00 usec
PLW1 5.00000000 W
F2 - Processing parameters
SI 65536
SF 500.1300000 MHz
WDW EM
SSB 0
LB 0.30 Hz
GB 0
PC 1.00
15 14 13 12 11 10 9 8 7 6 5 4 3 2 1 0 -1 -2 ppm
0.57
0.77
32.55
9.08
23.93
24.61

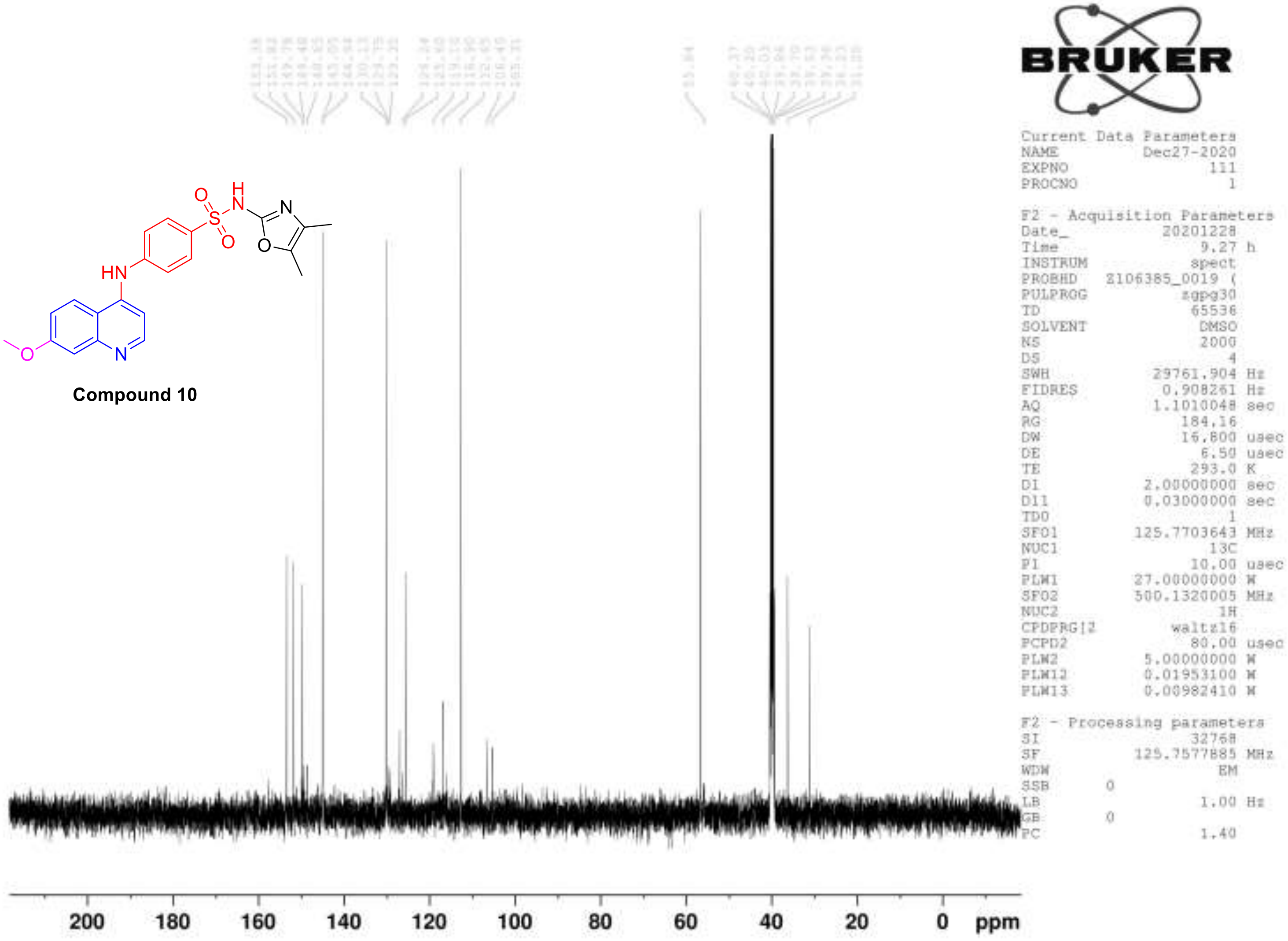
BRUKER
Current Data Parameters
NAME Dec27-2020
EXPNO 111
PROCNO 1
F2 - Acquisition Parameters
Date_ 20201228
Time 9.27 h
INSTRUM spect
PROBHD Z106385_0019 (
PULPROG zgpg30
TD 65536
SOLVENT DMSO
NS 2000
DS 4
SWH 29761.904 Hz
FIDRES 0.908261 Hz
AQ 1.1010048 sec
RG 184.16
DW 16.800 usec
DE 6.50 usec
TE 293.0 K
D1 2.00000000 sec
D11 0.03000000 sec
TD0 1
SFO1 125.7703643 MHz
NUC1 13C
P1 10.00 usec
PLW1 27.00000000 W
SFO2 500.1320005 MHz
NUC2 1H
CPDPRG[2 waltz16
PCPD2 80.00 usec
PLW2 5.00000000 W
PLW12 0.01953100 W
PLW13 0.00982410 W
F2 - Processing parameters
SI 32768
SF 125.7577885 MHz
WDW EM
SSB 0
LB 1.00 Hz
GB 0
PC 1.40
200 180 160 140 120 100 80 60 40 20 0 ppm

Compound 10

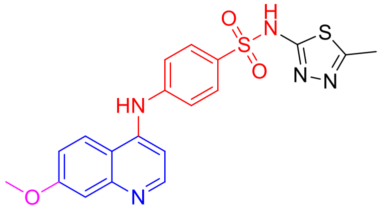

HN
H
N
O
S
O
S
N
N
N
O

Compound 11

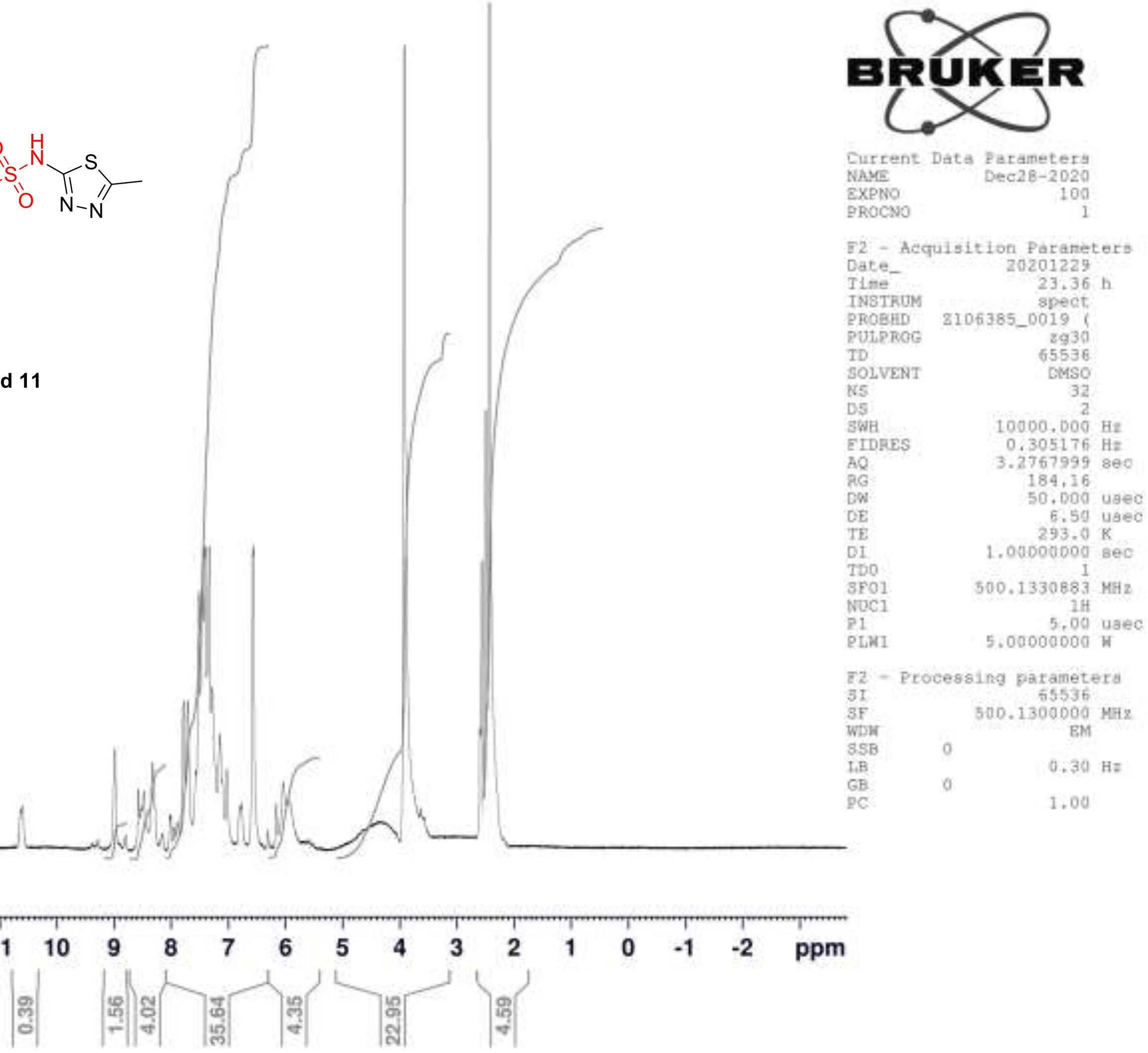

BRUKER
Current Data Parameters
NAME Dec28-2020
EXPNO 100
PROCNO 1
F2 - Acquisition Parameters
Date_ 20201229
Time 23.36 h
INSTRUM spect
PROBHD Z106385_0019 (
PULPROG zg30
TD 65536
SOLVENT DMSO
NS 32
DS 2
SWH 10000.000 Hz
FIDRES 0.305176 Hz
AQ 3.2767999 sec
RG 184.16
DW 50.000 usec
DE 6.50 usec
TE 293.0 K
D1 1.00000000 sec
TD0 1
SFO1 500.1330883 MHz
NUC1 1H
P1 5.00 usec
PLW1 5.00000000 W
F2 - Processing parameters
SI 65536
SF 500.1300000 MHz
WDW EM
SSB 0
LB 0.30 Hz
GB 0
PC 1.00
15 14 13 12 11 10 9 8 7 6 5 4 3 2 1 0 -1 -2 ppm
0.39
1.56
4.02
35.64
4.35
22.95
4.59

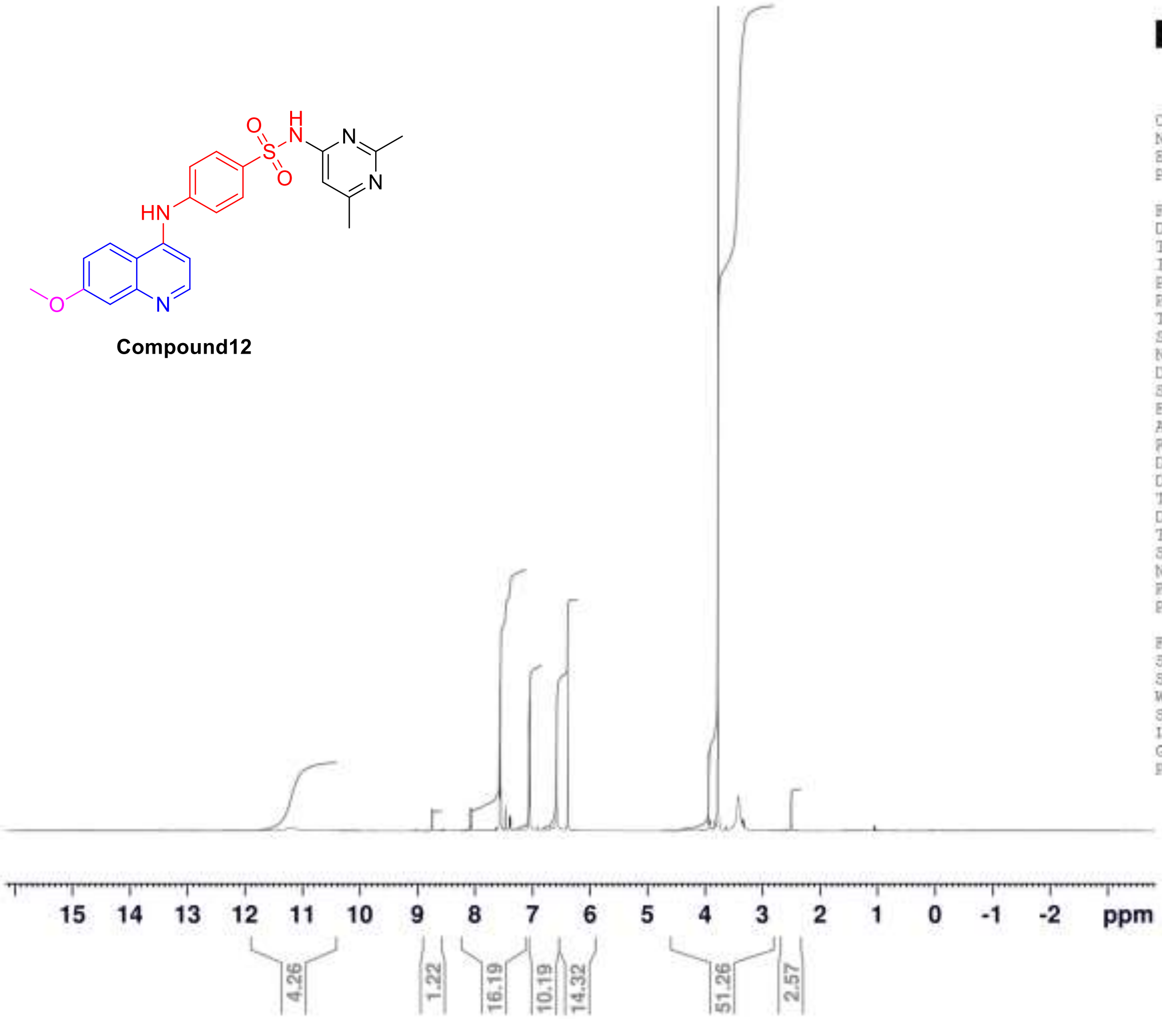

Compound12

BRUKER

Current Data Parameters

NAME	Dec28-2020
EXPNO	40
PROCNO	1

F2 - Acquisition Parameters

Date_	20201228
Time	19.30 h
INSTRUM	spect
PROBHD	Z106385_0019 (
PULPROG	zg30
TD	65536
SOLVENT	DMSO
NS	32
DS	2
SWH	10000.000 Hz
FIDRES	0.305176 Hz
AQ	3.2767999 sec
RG	184.16
DW	50.000 usec
DE	6.50 usec
TE	293.0 K
D1	1.00000000 sec
TD0	1
SFO1	500.1330883 MHz
NUC1	1H
P1	5.00 usec
PLW1	5.00000000 W

F2 - Processing parameters

SI	65536
SF	500.1300000 MHz
WDW	EM
SSB	0
LB	0.30 Hz
GB	0
PC	1.00

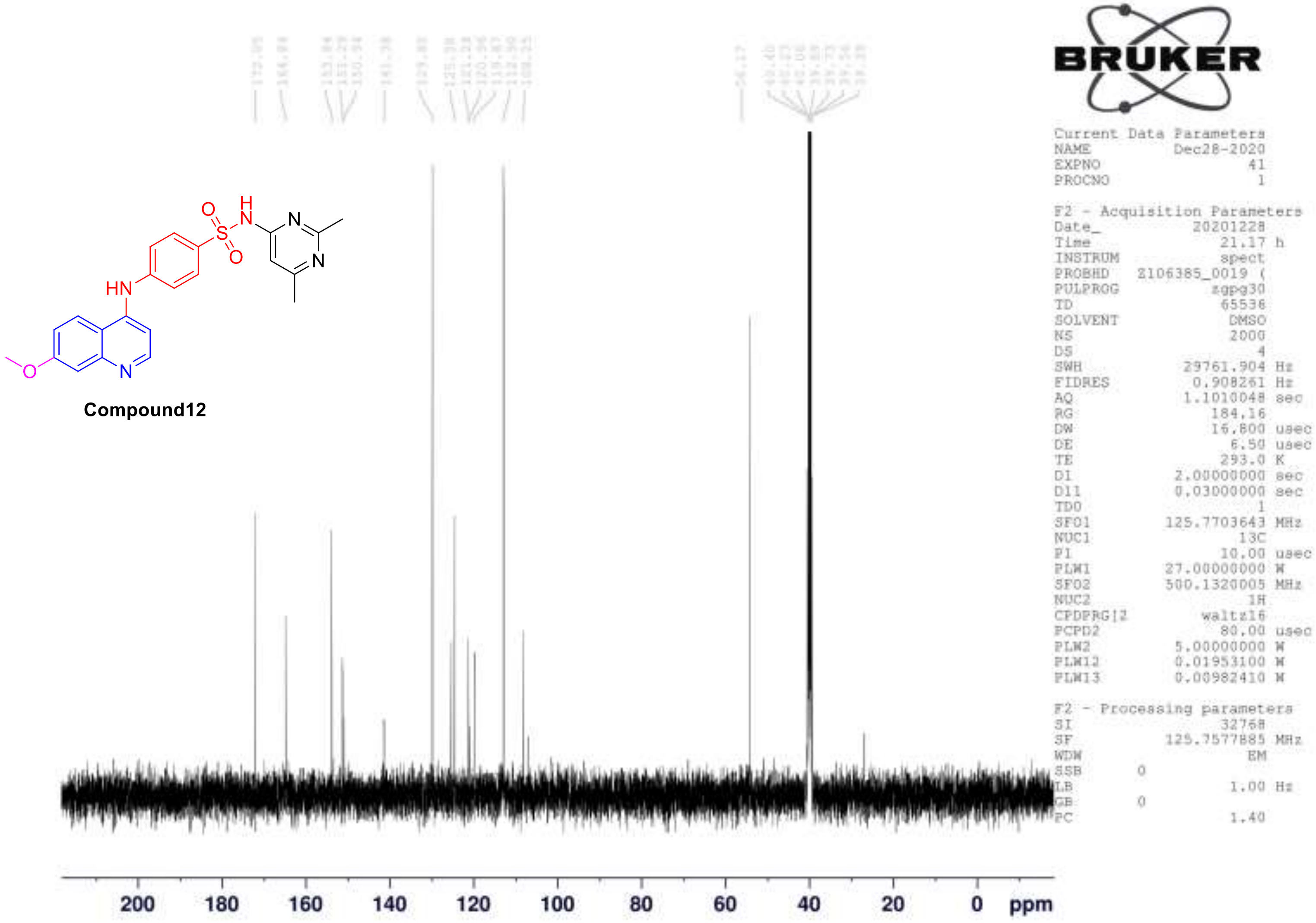
O
S
O
H
N
N
N
HN
O
N
Compound12
BRUKER
Current Data Parameters
NAME Dec28-2020
EXPNO 41
PROCNO 1
F2 - Acquisition Parameters
Date_ 20201228
Time 21.17 h
INSTRUM spect
PROBHD Z106385_0019 (
PULPROG zgpg30
TD 65536
SOLVENT DMSO
NS 2000
DS 4
SWH 29761.904 Hz
FIDRES 0.908261 Hz
AQ 1.1010048 sec
RG 184.16
DW 16.800 usec
DE 6.50 usec
TE 293.0 K
D1 2.00000000 sec
D11 0.03000000 sec
TD0 1
SFO1 125.7703643 MHz
NUC1 13C
P1 10.00 usec
PLW1 27.00000000 W
SFO2 500.1320005 MHz
NUC2 1H
CPDPRG[2 waltz16
PCPD2 80.00 usec
PLW2 5.00000000 W
PLW12 0.01953100 W
PLW13 0.00982410 W
F2 - Processing parameters
SI 32768
SF 125.7577885 MHz
WDW EM
SSB 0
LB 1.00 Hz
GB 0
PC 1.40
200
180
160
140
120
100
80
60
40
20
0
ppm

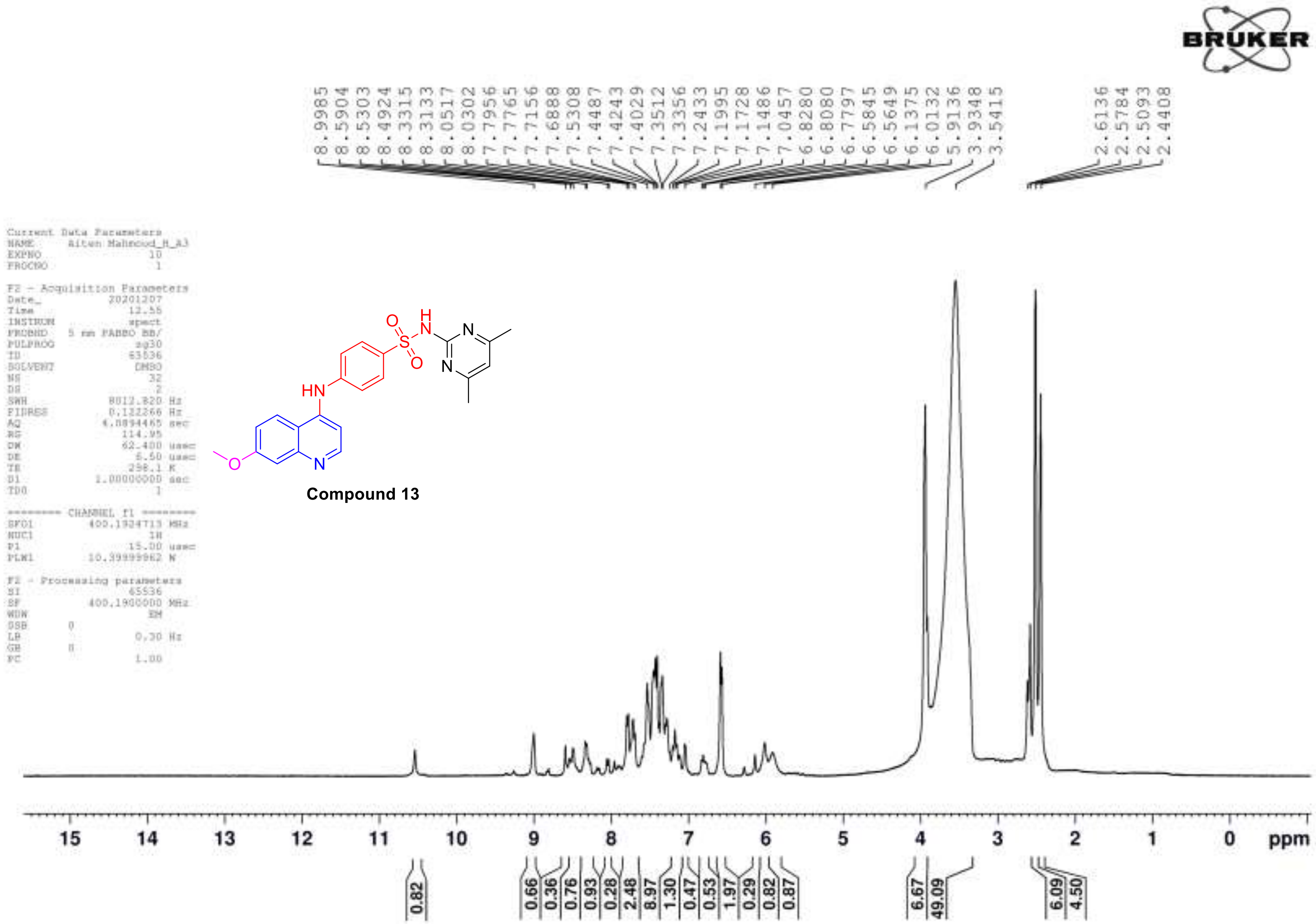

BRUKER
8.9985
8.5904
8.5303
8.4924
8.3315
8.3133
8.0517
8.0302
7.7956
7.7765
7.7156
7.6888
7.5308
7.4487
7.4243
7.4029
7.3512
7.3356
7.2433
7.1995
7.1728
7.1486
7.0457
6.8280
6.8080
6.7797
6.5845
6.5649
6.1375
6.0132
5.9136
3.9348
3.5415
2.6136
2.5784
2.5093
2.4408
Current Data Parameters
NAME Aiten Mahmoud_H_A3
EXPNO 10
PROCNO 1
F2 - Acquisition Parameters
Date_ 20201207
Time 12.55
INSTRUM spect
PROBHD 5 mm PABBO BB/
PULPROG zg30
TD 65536
SOLVENT DMSO
NS 32
DS 2
SWH 8012.820 Hz
FIDRES 0.122266 Hz
AQ 4.0894465 sec
RG 114.95
DW 62.400 usec
DE 6.50 usec
TE 298.1 K
D1 1.00000000 sec
TD0 1
======== CHANNEL f1 ========
SFO1 400.1924713 MHz
NUC1 1H
P1 15.00 usec
PLW1 10.39999962 W
F2 - Processing parameters
SI 65536
SF 400.1900000 MHz
WDW EM
SSB 0
LB 0.30 Hz
GB 0
PC 1.00
HN
O
S
O
H
N
N
N
N
O
15 14 13 12 11 10 9 8 7 6 5 4 3 2 1 0 ppm
0.82
0.66
0.36
0.76
0.93
0.28
2.48
8.97
1.30
0.47
0.53
1.97
0.29
0.82
0.87
6.67
49.09
6.09
4.50

Compound 13

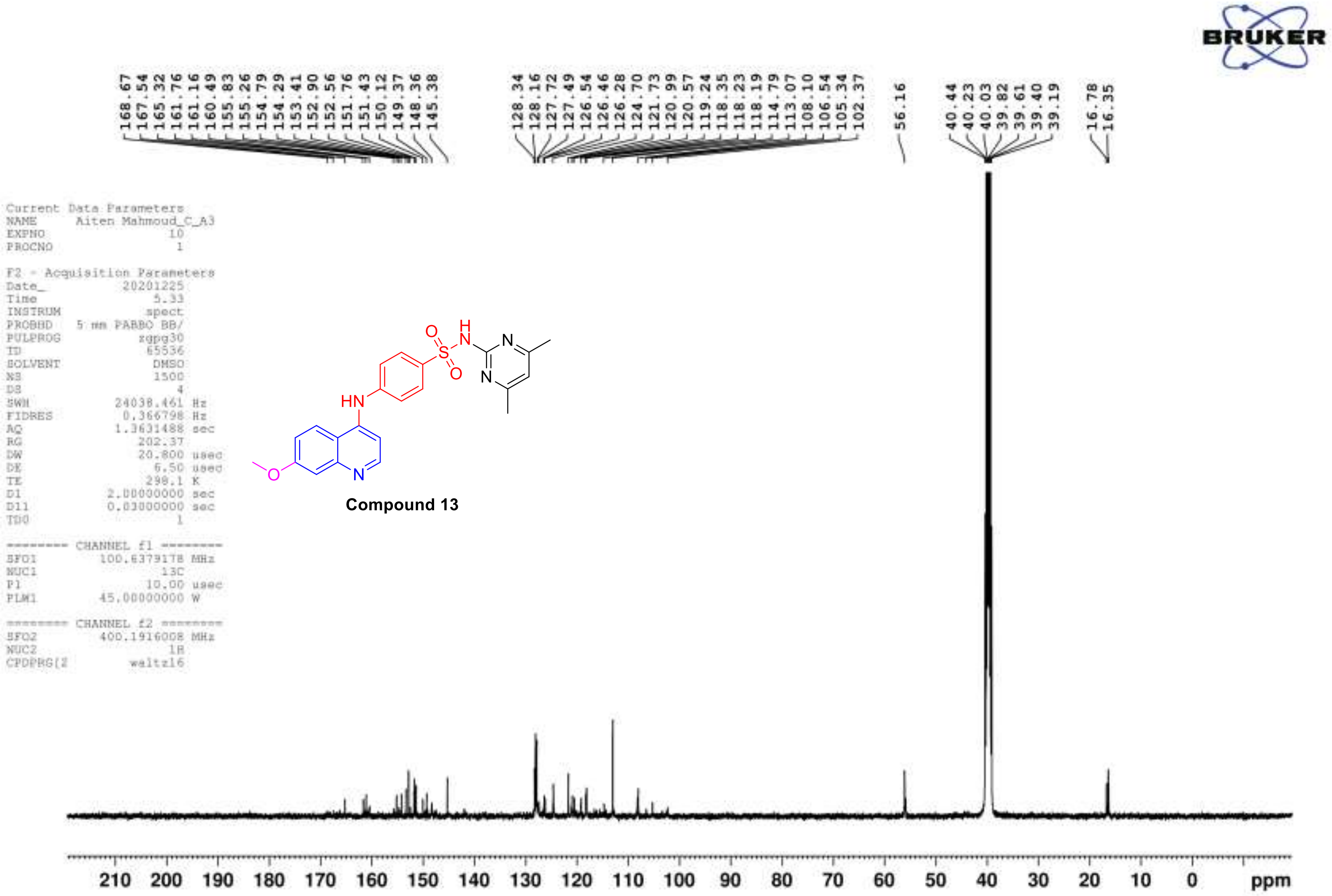

BRUKER
168.67
167.54
165.32
161.76
161.16
160.49
155.83
155.26
154.79
154.29
153.41
152.90
152.56
151.76
151.43
150.12
149.37
148.36
145.38
128.34
128.16
127.72
127.49
126.54
126.46
126.28
124.70
121.73
120.99
120.57
119.24
118.35
118.23
118.19
114.79
113.07
108.10
106.54
105.34
102.37
56.16
40.44
40.23
40.03
39.82
39.61
39.40
39.19
16.78
16.35
Current Data Parameters
NAME Aiten Mahmoud_C_A3
EXPNO 10
PROCNO 1
F2 - Acquisition Parameters
Date_ 20201225
Time 5.33
INSTRUM spect
PROBHD 5 mm PABBO BB/
PULPROG zgpg30
TD 65536
SOLVENT DMSO
NS 1500
DS 4
SWH 24038.461 Hz
FIDRES 0.366798 Hz
AQ 1.3631488 sec
RG 202.37
DW 20.800 usec
DE 6.50 usec
TE 298.1 K
D1 2.00000000 sec
D11 0.03000000 sec
TD0 1
======== CHANNEL f1 ========
SFO1 100.6379178 MHz
NUC1 13C
P1 10.00 usec
PLW1 45.00000000 W
======== CHANNEL f2 ========
SFO2 400.1916008 MHz
NUC2 1H
CPDPRG[2 waltz16
Compound 13
210 200 190 180 170 160 150 140 130 120 110 100 90 80 70 60 50 40 30 20 10 0 ppm

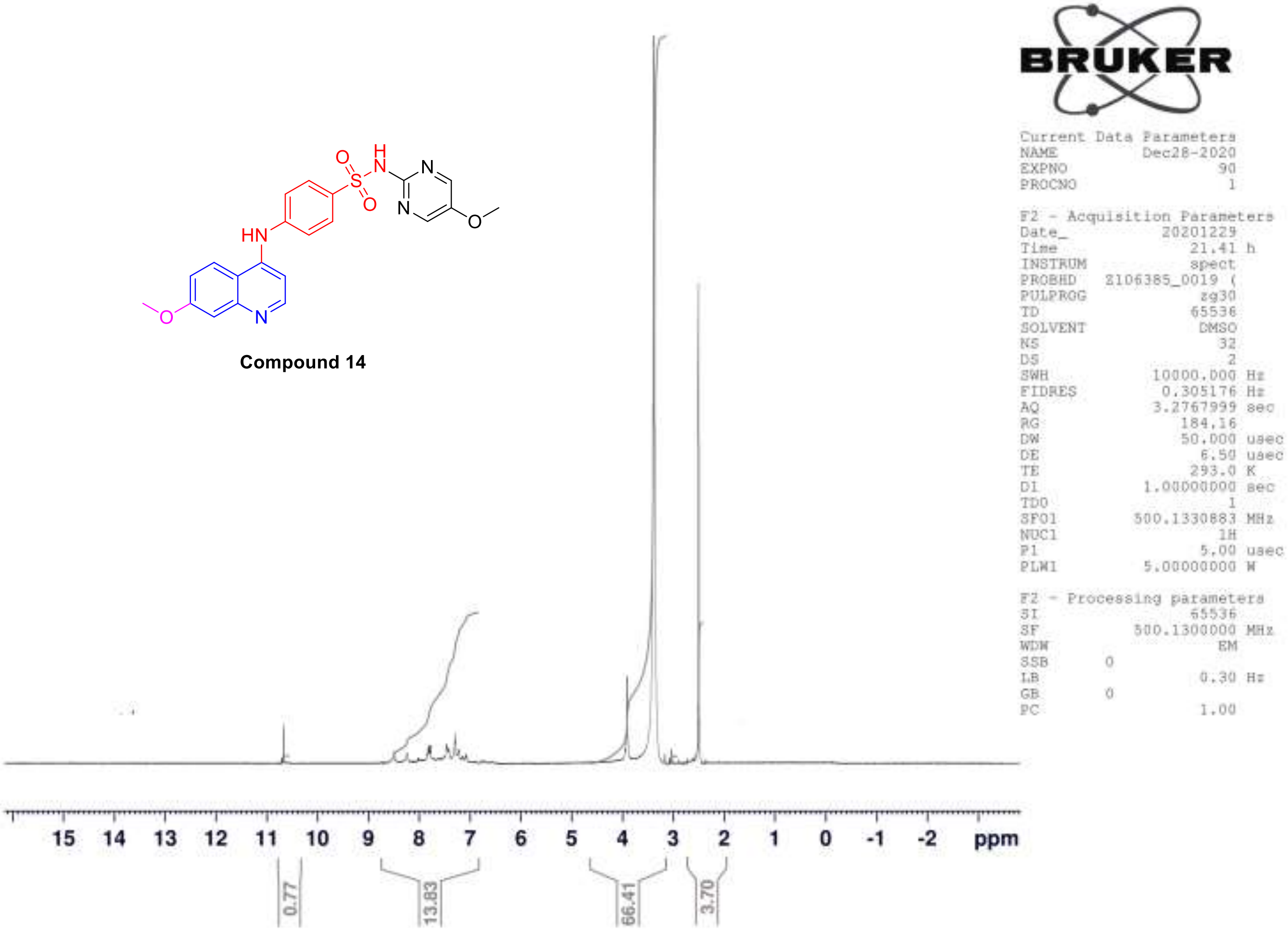
O
H
N
N
N
O
S
O
HN
O
N
Compound 14
BRUKER
Current Data Parameters
NAME Dec28-2020
EXPNO 90
PROCNO 1
F2 - Acquisition Parameters
Date_ 20201229
Time 21.41 h
INSTRUM spect
PROBHD Z106385_0019 (
PULPROG zg30
TD 65536
SOLVENT DMSO
NS 32
DS 2
SWH 10000.000 Hz
FIDRES 0.305176 Hz
AQ 3.2767999 sec
RG 184.16
DW 50.000 usec
DE 6.50 usec
TE 293.0 K
D1 1.00000000 sec
TD0 1
SFO1 500.1330883 MHz
NUC1 1H
P1 5.00 usec
PLW1 5.00000000 W
F2 - Processing parameters
SI 65536
SF 500.1300000 MHz
WDW EM
SSB 0
LB 0.30 Hz
GB 0
PC 1.00
15 14 13 12 11 10 9 8 7 6 5 4 3 2 1 0 -1 -2 ppm
0.77
13.83
66.41
3.70

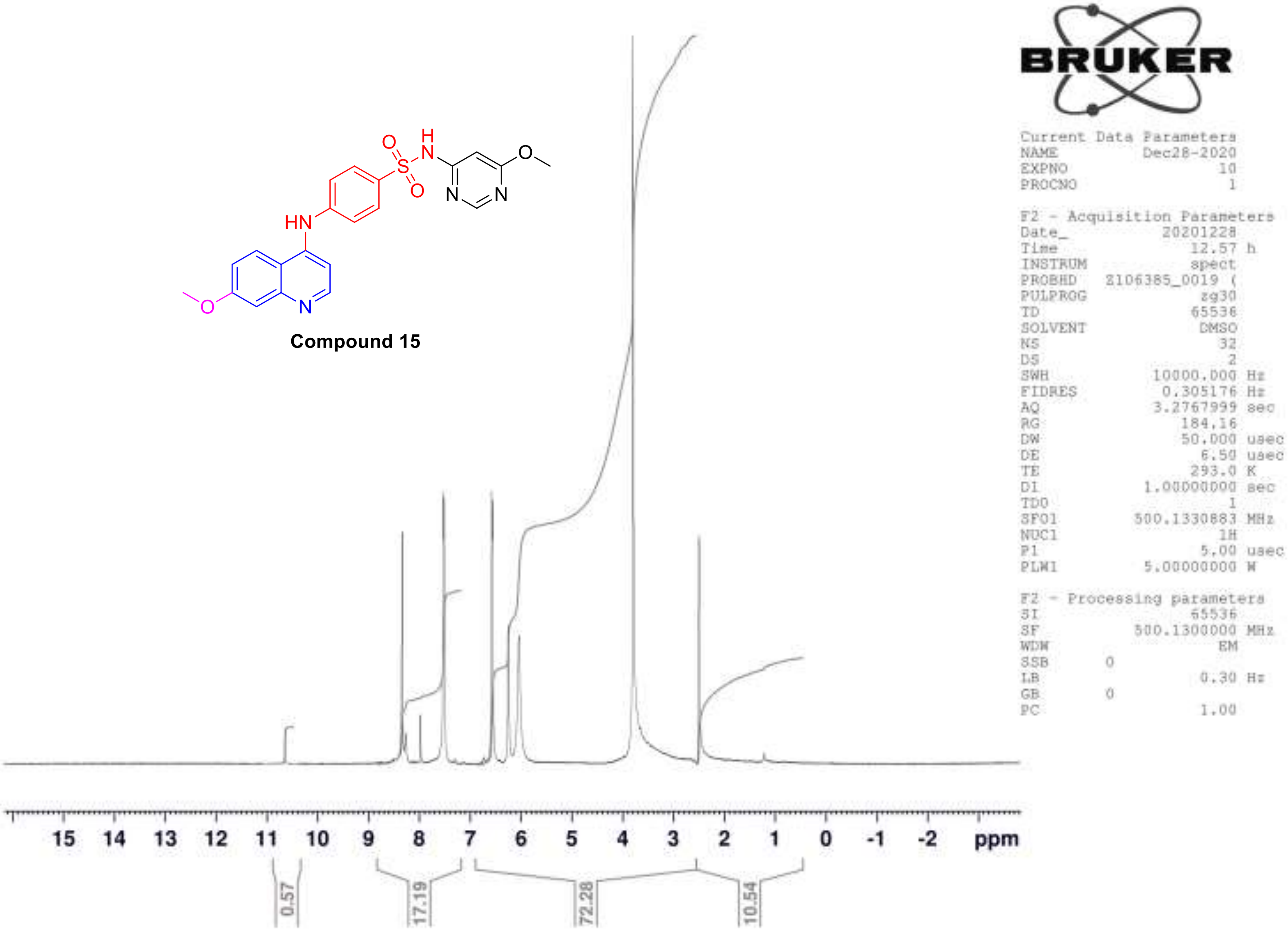

BRUKER
Current Data Parameters
NAME Dec28-2020
EXPNO 10
PROCNO 1
F2 - Acquisition Parameters
Date_ 20201228
Time 12.57 h
INSTRUM spect
PROBHD Z106385_0019 (
PULPROG zg30
TD 65536
SOLVENT DMSO
NS 32
DS 2
SWH 10000.000 Hz
FIDRES 0.305176 Hz
AQ 3.2767999 sec
RG 184.16
DW 50.000 usec
DE 6.50 usec
TE 293.0 K
D1 1.00000000 sec
TD0 1
SFO1 500.1330883 MHz
NUC1 1H
P1 5.00 usec
PLW1 5.00000000 W
F2 - Processing parameters
SI 65536
SF 500.1300000 MHz
WDW EM
SSB 0
LB 0.30 Hz
GB 0
PC 1.00
15 14 13 12 11 10 9 8 7 6 5 4 3 2 1 0 -1 -2 ppm
0.57
17.19
72.28
10.54

Compound 15

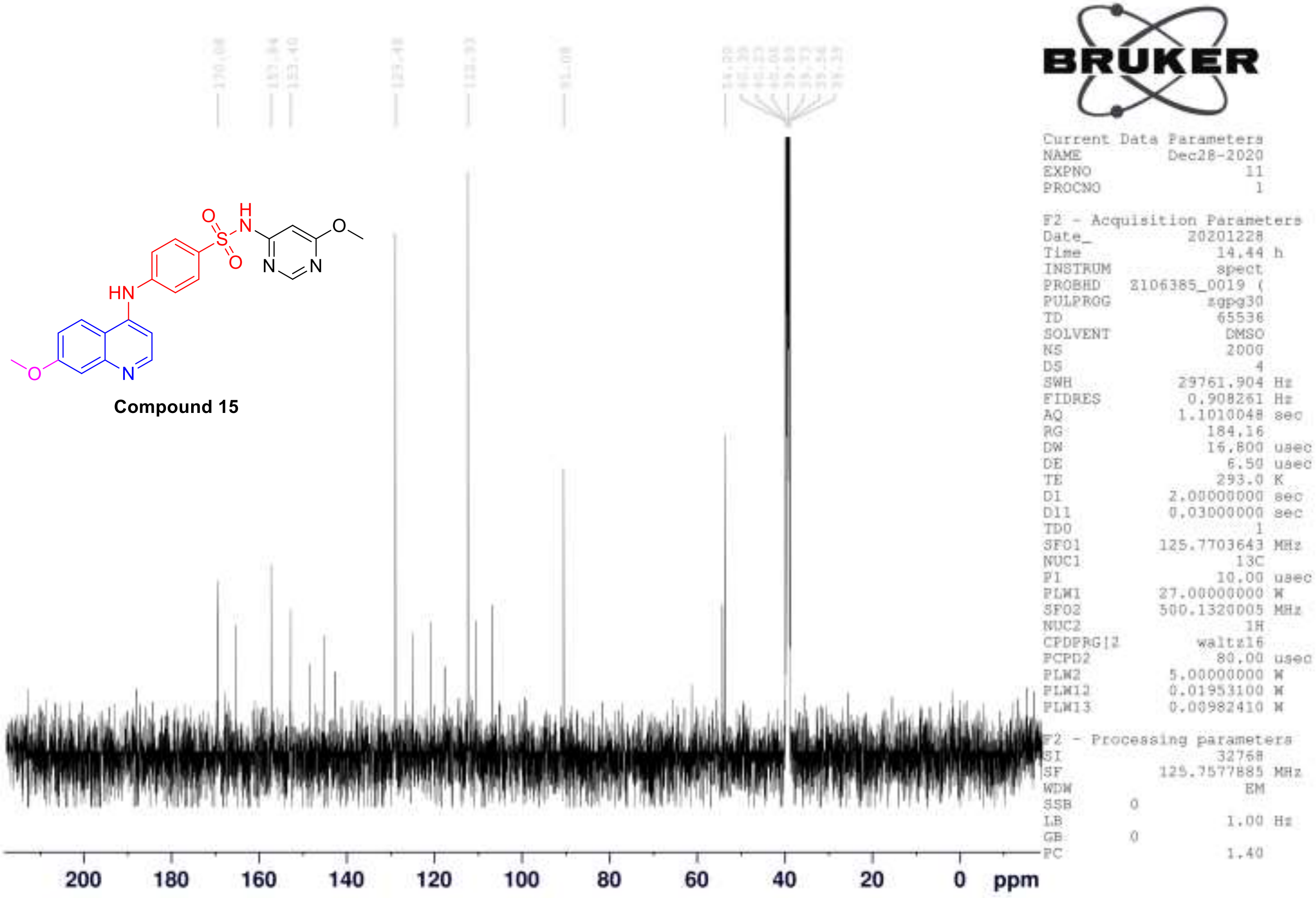
Compound 15
BRUKER
Current Data Parameters
NAME Dec28-2020
EXPNO 11
PROCNO 1
F2 - Acquisition Parameters
Date_ 20201228
Time 14.44 h
INSTRUM spect
PROBHD Z106385_0019 (
PULPROG zgpg30
TD 65536
SOLVENT DMSO
NS 2000
DS 4
SWH 29761.904 Hz
FIDRES 0.908261 Hz
AQ 1.1010048 sec
RG 184.16
DW 16.800 usec
DE 6.50 usec
TE 293.0 K
D1 2.00000000 sec
D11 0.03000000 sec
TD0 1
SFO1 125.7703643 MHz
NUC1 13C
P1 10.00 usec
PLW1 27.00000000 W
SFO2 500.1320005 MHz
NUC2 1H
CPDPRG[2 waltz16
PCPD2 80.00 usec
PLW2 5.00000000 W
PLW12 0.01953100 W
PLW13 0.00982410 W
F2 - Processing parameters
SI 32768
SF 125.7577885 MHz
WDW EM
SSB 0
LB 1.00 Hz
GB 0
PC 1.40
200 180 160 140 120 100 80 60 40 20 0 ppm

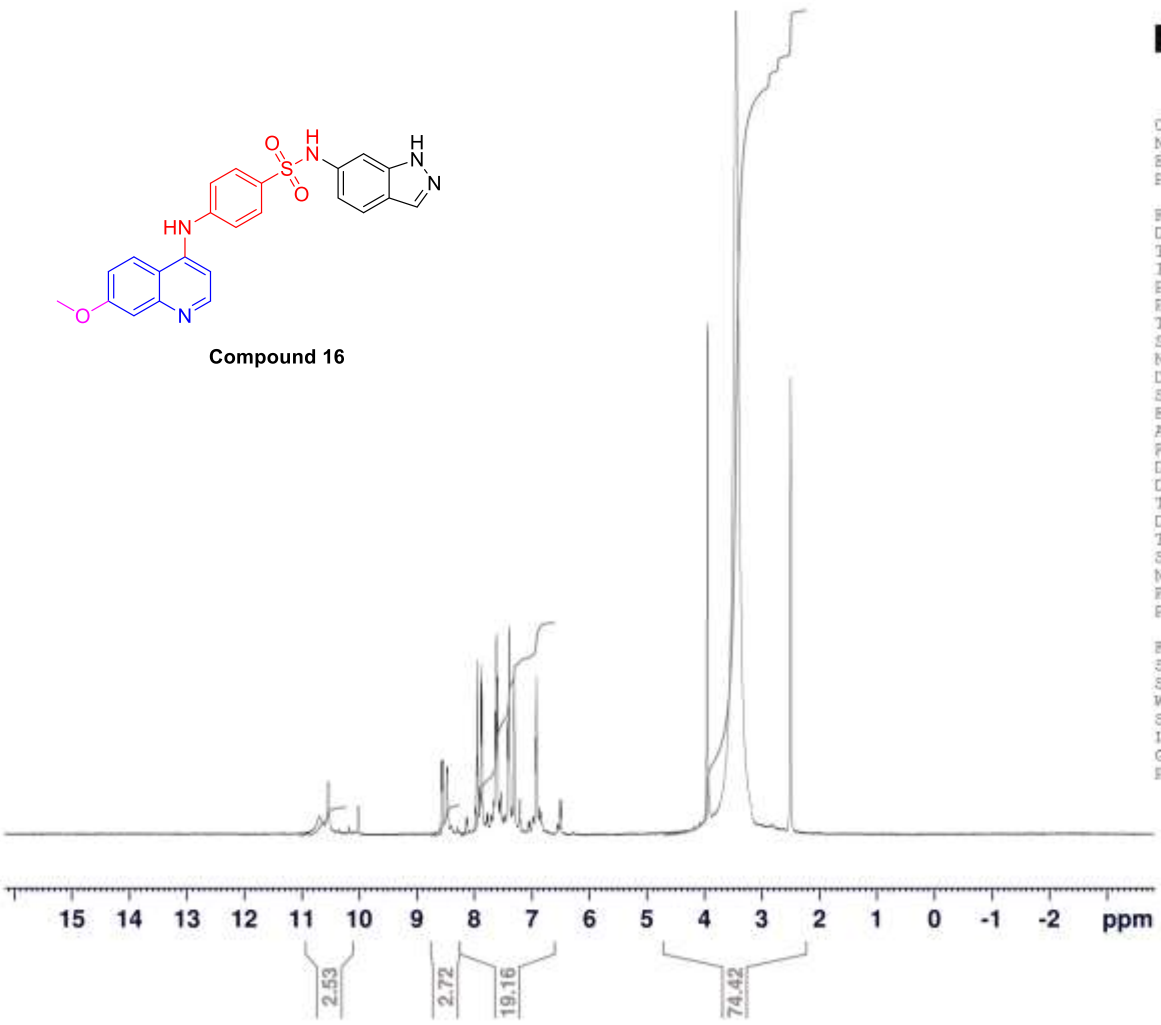

Compound 16

BRUKER

Current Data Parameters
NAME Dec28-2020
EXPNO 70
PROCNO 1

F2 - Acquisition Parameters
Date_ 20201229
Time 17.01 h
INSTRUM spect
PROBHD Z106385_0019 (
PULPROG zg30
TD 65536
SOLVENT DMSO
NS 32
DS 2
SWH 10000.000 Hz
FIDRES 0.305176 Hz
AQ 3.2767999 sec
RG 184.16
DW 50.000 usec
DE 6.50 usec
TE 293.0 K
D1 1.00000000 sec
TD0 1
SFO1 500.1330883 MHz
NUC1 1H
P1 5.00 usec
PLW1 5.00000000 W

F2 - Processing parameters
SI 65536
SF 500.1300000 MHz
WDW EM
SSB 0
LB 0.30 Hz
GB 0
PC 1.00

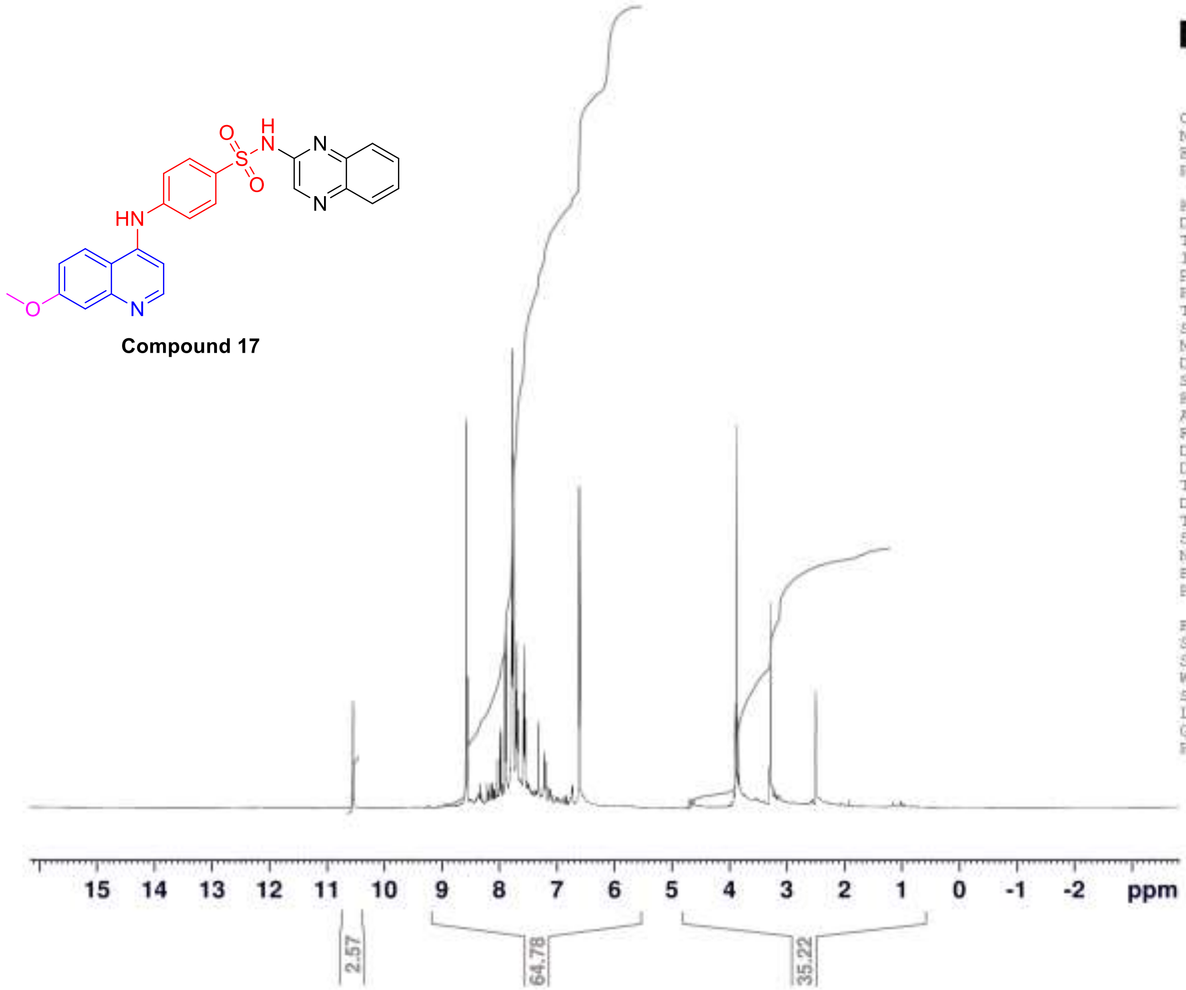

Compound 17


```
Current Data Parameters
NAME              Dec27-2020
EXPNO                     80
PROCNO                     1

F2 - Acquisition Parameters
Date_               20201228
Time                  1.59 h
INSTRUM                spect
PROBHD        Z106385_0019 (
PULPROG                 zg30
TD                     65536
SOLVENT                 DMSO
NS                        32
DS                         2
SWH               10000.000 Hz
FIDRES             0.305176 Hz
AQ                3.2767999 sec
RG                   131.78
DW                   50.000 usec
DE                     6.50 usec
TE                    293.0 K
D1               1.00000000 sec
TD0                        1
SFO1            500.1330883 MHz
NUC1                      1H
P1                     5.00 usec
PLW1             5.00000000 W

F2 - Processing parameters
SI                     65536
SF              500.1300000 MHz
WDW                       EM
SSB      0
LB                     0.30 Hz
GB       0
PC                     1.00
```

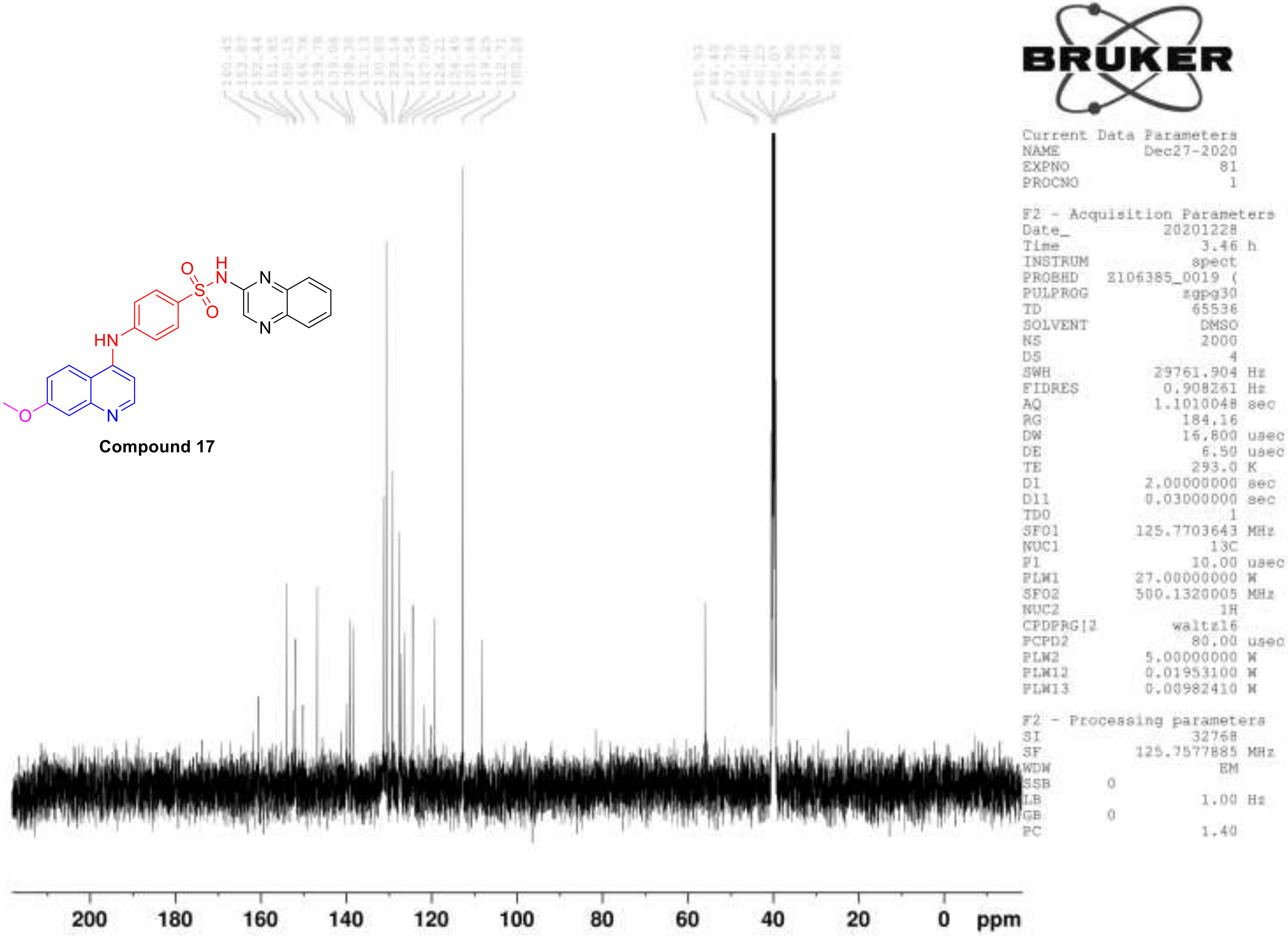

Compound 17

Current Data Parameters
NAME Dec27-2020
EXPNO 81
PROCNO 1

F2 - Acquisition Parameters
Date_ 20201228
Time 3.46 h
INSTRUM spect
PROBHD Z106385_0019 (
PULPROG zgpg30
TD 65536
SOLVENT DMSO
NS 2000
DS 4
SWH 29761.904 Hz
FIDRES 0.908261 Hz
AQ 1.1010048 sec
RG 184.16
DW 16.800 usec
DE 6.50 usec
TE 293.0 K
D1 2.00000000 sec
D11 0.03000000 sec
TD0 1
SFO1 125.7703643 MHz
NUC1 13C
P1 10.00 usec
PLW1 27.00000000 W
SFO2 500.1320005 MHz
NUC2 1H
CPDPRG[2 waltz16
PCPD2 80.00 usec
PLW2 5.00000000 W
PLW12 0.01953100 W
PLW13 0.00982410 W

F2 - Processing parameters
SI 32768
SF 125.7577885 MHz
WDW EM
SSB 0
LB 1.00 Hz
GB 0
PC 1.40

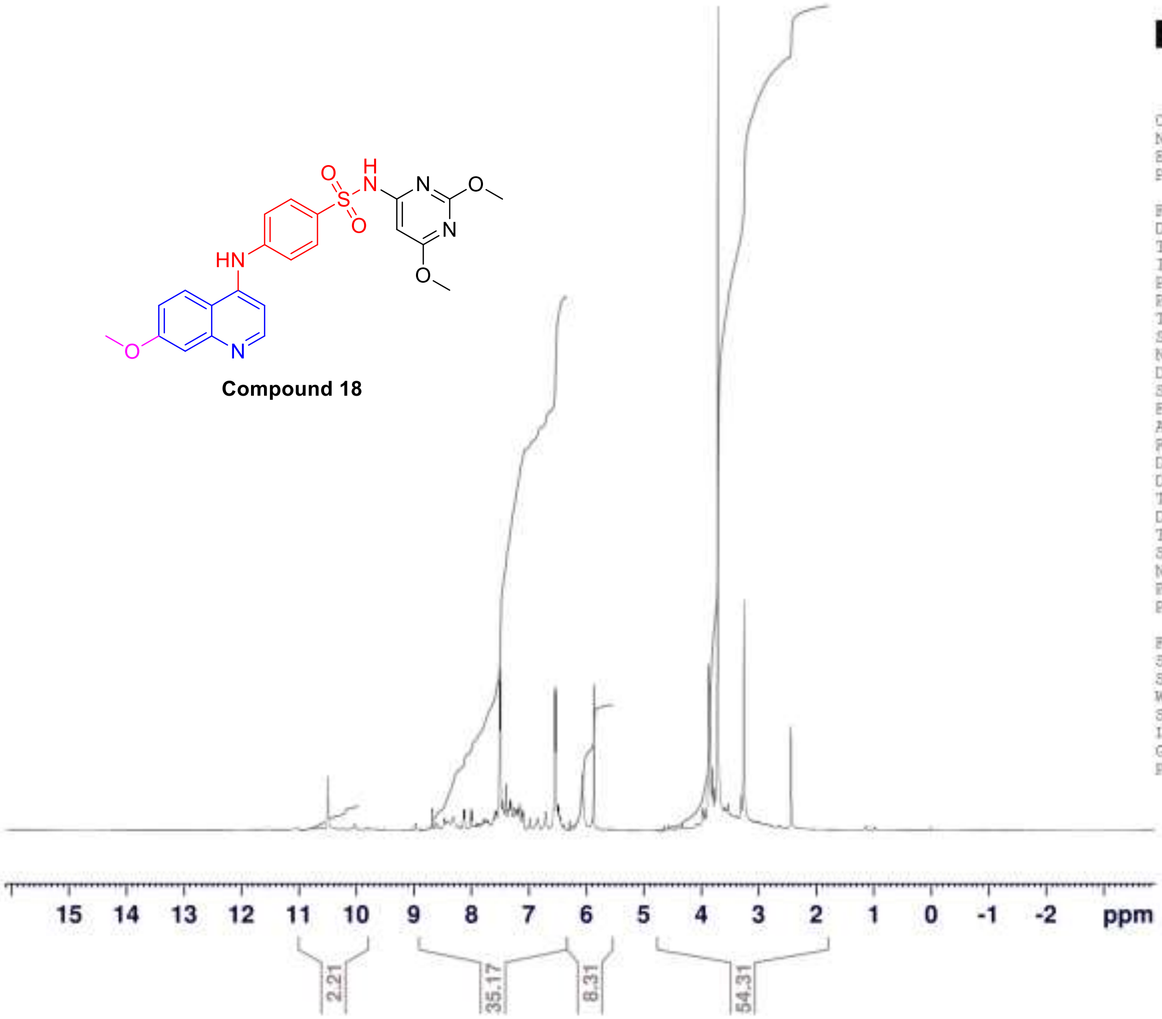

Compound 18

BRUKER

Current Data Parameters
NAME Dec27-2020
EXPNO 90
PROCNO 1

F2 - Acquisition Parameters
Date_ 20201228
Time 3.53 h
INSTRUM spect
PROBHD Z106385_0019 (
PULPROG zg30
TD 65536
SOLVENT DMSO
NS 32
DS 2
SWH 10000.000 Hz
FIDRES 0.305176 Hz
AQ 3.2767999 sec
RG 144.96
DW 50.000 usec
DE 6.50 usec
TE 293.0 K
D1 1.00000000 sec
TD0 1
SFO1 500.1330883 MHz
NUC1 1H
P1 5.00 usec
PLW1 5.00000000 W

F2 - Processing parameters
SI 65536
SF 500.1300339 MHz
WDW EM
SSB 0
LB 0.30 Hz
GB 0
PC 1.00

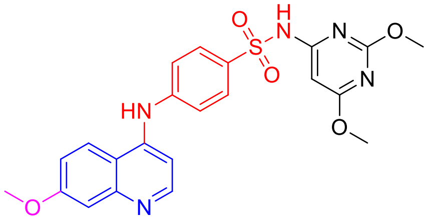
O
O
S
H
N
N
N
O
O
HN
O
N

Compound 18

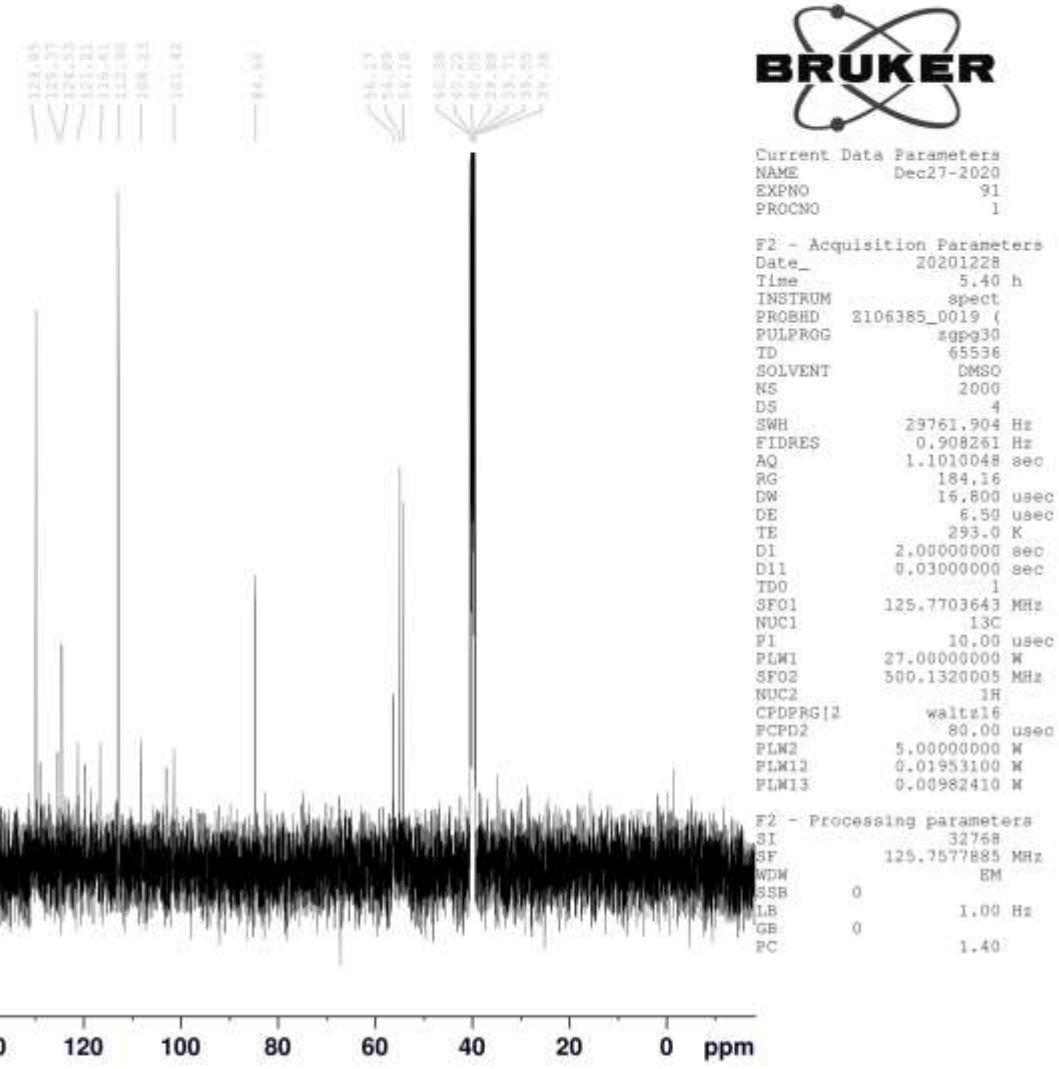
BRUKER
Current Data Parameters
NAME Dec27-2020
EXPNO 91
PROCNO 1
F2 - Acquisition Parameters
Date_ 20201228
Time 5.40 h
INSTRUM spect
PROBHD Z106385_0019 (
PULPROG zgpg30
TD 65536
SOLVENT DMSO
NS 2000
DS 4
SWH 29761.904 Hz
FIDRES 0.908261 Hz
AQ 1.1010048 sec
RG 184.16
DW 16.800 usec
DE 6.50 usec
TE 293.0 K
D1 2.00000000 sec
D11 0.03000000 sec
TD0 1
SFO1 125.7703643 MHz
NUC1 13C
P1 10.00 usec
PLW1 27.00000000 W
SFO2 500.1320005 MHz
NUC2 1H
CPDPRG[2 waltz16
PCPD2 80.00 usec
PLW2 5.00000000 W
PLW12 0.01953100 W
PLW13 0.00982410 W
F2 - Processing parameters
SI 32768
SF 125.7577885 MHz
WDW EM
SSB 0
LB 1.00 Hz
GB 0
PC 1.40
200
180
160
140
120
100
80
60
40
20
0
ppm

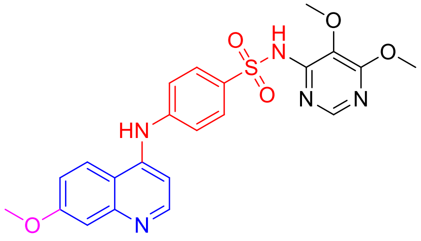

Compound 19

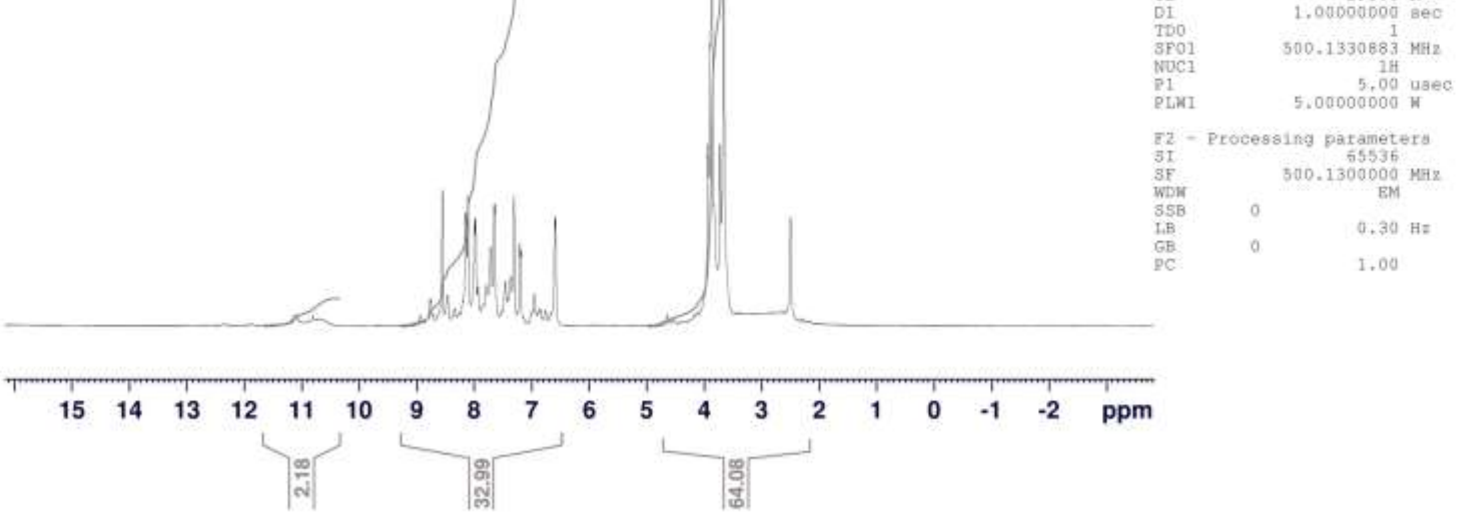

BRUKER
Current Data Parameters
NAME Dec27-2020
EXPNO 100
PROCNO 1
F2 - Acquisition Parameters
Date_ 20201228
Time 5.47 h
INSTRUM spect
PROBHD Z106385_0019 (
PULPROG zg30
TD 65536
SOLVENT DMSO
NS 32
DS 2
SWH 10000.000 Hz
FIDRES 0.305176 Hz
AQ 3.2767999 sec
RG 102.86
DW 50.000 usec
DE 6.50 usec
TE 293.0 K
D1 1.00000000 sec
TD0 1
SFO1 500.1330883 MHz
NUC1 1H
P1 5.00 usec
PLW1 5.00000000 W
F2 - Processing parameters
SI 65536
SF 500.1300000 MHz
WDW EM
SSB 0
LB 0.30 Hz
GB 0
PC 1.00
15 14 13 12 11 10 9 8 7 6 5 4 3 2 1 0 -1 -2 ppm
2.18
32.99
64.08

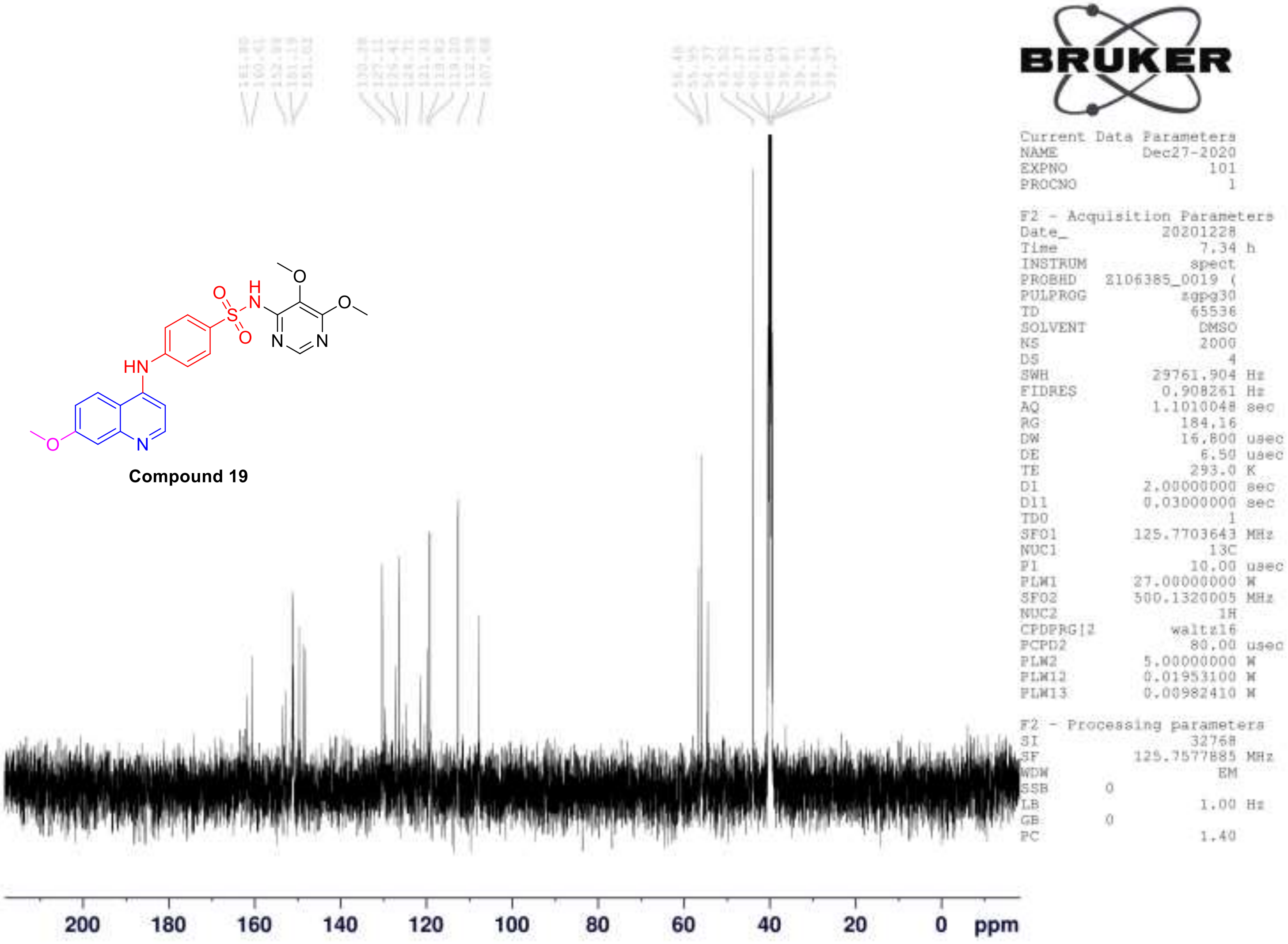
Compound 19
BRUKER
Current Data Parameters
NAME Dec27-2020
EXPNO 101
PROCNO 1
F2 - Acquisition Parameters
Date_ 20201228
Time 7.34 h
INSTRUM spect
PROBHD Z106385_0019 (
PULPROG zgpg30
TD 65536
SOLVENT DMSO
NS 2000
DS 4
SWH 29761.904 Hz
FIDRES 0.908261 Hz
AQ 1.1010048 sec
RG 184.16
DW 16.800 usec
DE 6.50 usec
TE 293.0 K
D1 2.00000000 sec
D11 0.03000000 sec
TD0 1
SFO1 125.7703643 MHz
NUC1 13C
P1 10.00 usec
PLW1 27.00000000 W
SFO2 500.1320005 MHz
NUC2 1H
CPDPRG[2 waltz16
PCPD2 80.00 usec
PLW2 5.00000000 W
PLW12 0.01953100 W
PLW13 0.00982410 W
F2 - Processing parameters
SI 32768
SF 125.7577885 MHz
WDW EM
SSB 0
LB 1.00 Hz
GB 0
PC 1.40
200 180 160 140 120 100 80 60 40 20 0 ppm

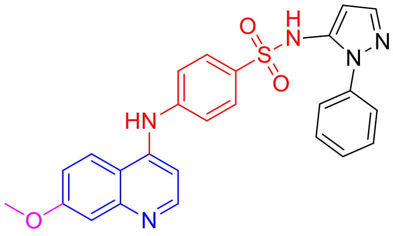

Compound 20

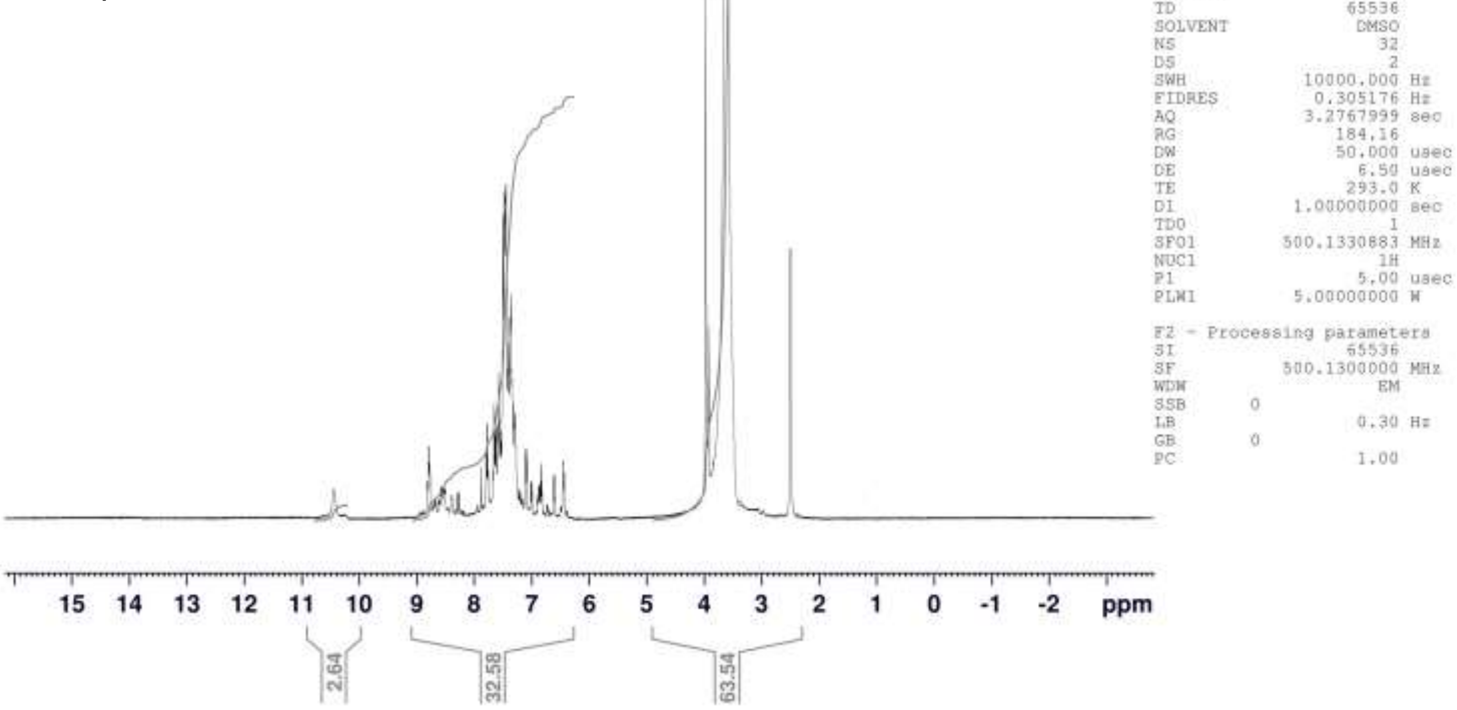
Current Data Parameters
NAME Dec28-2020
EXPNO 60
PROCNO 1
F2 - Acquisition Parameters
Date_ 20201229
Time 14.15 h
INSTRUM spect
PROBHD Z106385_0019 (
PULPROG zg30
TD 65536
SOLVENT DMSO
NS 32
DS 2
SWH 10000.000 Hz
FIDRES 0.305176 Hz
AQ 3.2767999 sec
RG 184.16
DW 50.000 usec
DE 6.50 usec
TE 293.0 K
D1 1.00000000 sec
TD0 1
SFO1 500.1330883 MHz
NUC1 1H
P1 5.00 usec
PLW1 5.00000000 W
F2 - Processing parameters
SI 65536
SF 500.1300000 MHz
WDW EM
SSB 0
LB 0.30 Hz
GB 0
PC 1.00
15 14 13 12 11 10 9 8 7 6 5 4 3 2 1 0 -1 -2 ppm
2.64
32.58
63.54
